# Rigid crosslinking of the CD3 complex leads to superior T cell stimulation

**DOI:** 10.3389/fimmu.2024.1434463

**Published:** 2024-08-30

**Authors:** Alfreda D. Nelson, Liangyu Wang, Kimberly G. Laffey, Laura R. E. Becher, Christopher A. Parks, Michele M. Hoffmann, Belinda K. Galeano, Ashutosh Mangalam, Emma Teixeiro, Tommi A. White, Adam G. Schrum, John F. Cannon, Diana Gil

**Affiliations:** ^1^ Department of Surgery, School of Medicine, University of Missouri, Columbia, MO, United States; ^2^ Department of Molecular Microbiology and Immunology, University of Missouri, Columbia, MO, United States; ^3^ Department of Immunology, Mayo Clinic College of Medicine and Science, Rochester, MN, United States; ^4^ Department of Pathology, University of Iowa, Iowa City, IA, United States; ^5^ Department of Biochemistry, University of Missouri, Columbia, MO, United States; ^6^ Department of Biomedical, Biological and Medical Engineering, College of Engineering, University of Missouri, Columbia, MO, United States

**Keywords:** T cell receptor engagement and triggering, antibody fragment structure, CD3/antibody crosslinking, T cell division and apoptosis, anti-CD3 Fab-based therapies, EAE (experimental autoimmune encephalomyelitis), molecular dynamic simulation

## Abstract

Functionally bivalent non-covalent Fab dimers (Bi-Fabs) specific for the TCR/CD3 complex promote CD3 signaling on T cells. While comparing functional responses to stimulation with Bi-Fab, F(ab’)2 or mAb specific for the same CD3 epitope, we observed fratricide requiring anti-CD3 bridging of adjacent T cells. Surprisingly, anti-CD3 Bi-Fab ranked first in fratricide potency, followed by anti-CD3 F(ab’)2 and anti-CD3 mAb. Low resolution structural studies revealed anti-CD3 Bi-Fabs and F(ab’)2 adopt similar global shapes with CD3-binding sites oriented outward. However, under molecular dynamic simulations, anti-CD3 Bi-Fabs crosslinked CD3 more rigidly than F(ab’)2. Furthermore, molecular modelling of Bi-Fab and F(ab’)2 binding to CD3 predicted crosslinking of T cell antigen receptors located in opposing plasma membrane domains, a feature fitting with T cell fratricide observed. Thus, increasing rigidity of Fab-CD3 crosslinking between opposing effector-target pairs may result in stronger T cell effector function. These findings could guide improving clinical performance of bi-specific anti-CD3 drugs.

## Introduction

Anti-CD3 bi-specific antibodies (anti-CD3 BiAbs) are recombinant antibody-based hybrid molecules of dual specificity designed to enable the clinical application of a concept that emerged in the field in the 1980s: to re-direct T cells against any cell expressing antigens of choice bypassing MHC restricted TCR antigen recognition. These hybrid antibody molecules of dual specificity are designed for bridging T cells to undesired cells such as tumor cells, to aid the clearance of the latter (1, 2).

It took decades, but in 2014, the first anti-CD3 BiAb, blinatumomab (Blincyto; Amgen, Inc.) was approved by the FDA for treatment of Philadelphia chromosome-negative relapsed or refractory precursor B-cell acute lymphoblastic leukemia (R/R ALL) (3). Blinatumomab targets the B cell marker CD19 to redirect T cells against malignant B cells. Blinatumomab is a bispecific T-cell engager (BiTE) composed of two single-chain variable fragments (scFvs) that bind respectively the B and T cell markers CD19 and CD3ε, which are connected by a flexible linker in the absence of an Fc portion.

Fueled by the success of Blinatumomab in the clinic, the field is intensively developing additional anti-CD3 BiAbs against not only hematological malignancies, but solid tumors as well (4, 5). Three anti-CD3 BiAbs have been FDA approved recently to treat lymphomas (Glofitamab, Epcoritamab and Mosumetuzumab) (6) solidifying progress of BiAbs usage against blood malignancies (6). In addition, Tebentafusp, a BiAbs linking T cells to APCs presenting HLA-A2/gp100 antigens, was FDA approved in 2022 as the first anti-CD3 BiAb to treat non-lymphoma malignances such as advanced uveal melanoma (7).

Regardless of anti-CD3 BiAbs reaching the clinic, several limitations of these therapies to treat blood and solid cancers remain unsolved. First, anti-CD3 BiAbs may bridge T cells to healthy cells that express the antigen selected to target the tumor causing to “on target off tumor” side effects (6, 8). Additionally, CD3 crosslinking capacity of BiAbs can lead to over stimulation of T cells, which in turn may cause cytokine release syndrome, and neurological toxicities (9, 10). Anti-CD3 BiAbs share these mechanisms of toxicity mediated by activation of immune function with other immunotherapeutic approaches like CAR T cells (4). On the other hand, poor immunogenicity of tumor microenvironment, and/or presence of physical barriers around the tumor, may limit recruitment of T cells and by extension effectiveness of anti-CD3 BiAbs designed against solid tumors (11, 12). Finally, the capacity of the T cells to robustly respond to the stimulation caused by TCR/CD3 complex crosslinking when T cell bound anti-CD3 BiAbs bind to the tumor cells may be reduced due to exhaustion and other immunosuppressive mechanisms exerted by the tumor and its microenvironment (12, 13).

Given the clinical potential yet to be achieved, anti-CD3 BiAbs shortcomings described above are currently subjected to intense investigation, especially those limiting efficacy of anti-CD3 BiAbs targeting solid malignancies for which clinical success is modest (8, 11, 12). However, understanding TCR/CD3 triggering mechanisms utilized by these drugs remains unaddressed, with only limited studies describing CD3 signaling events leading to robust effector responses from T cells (14, 15). Yet, insight into such mechanisms to initiate downstream signaling could also lead to optimization of anti-CD3 BiAbs clinical performance.

**Figure 1:**
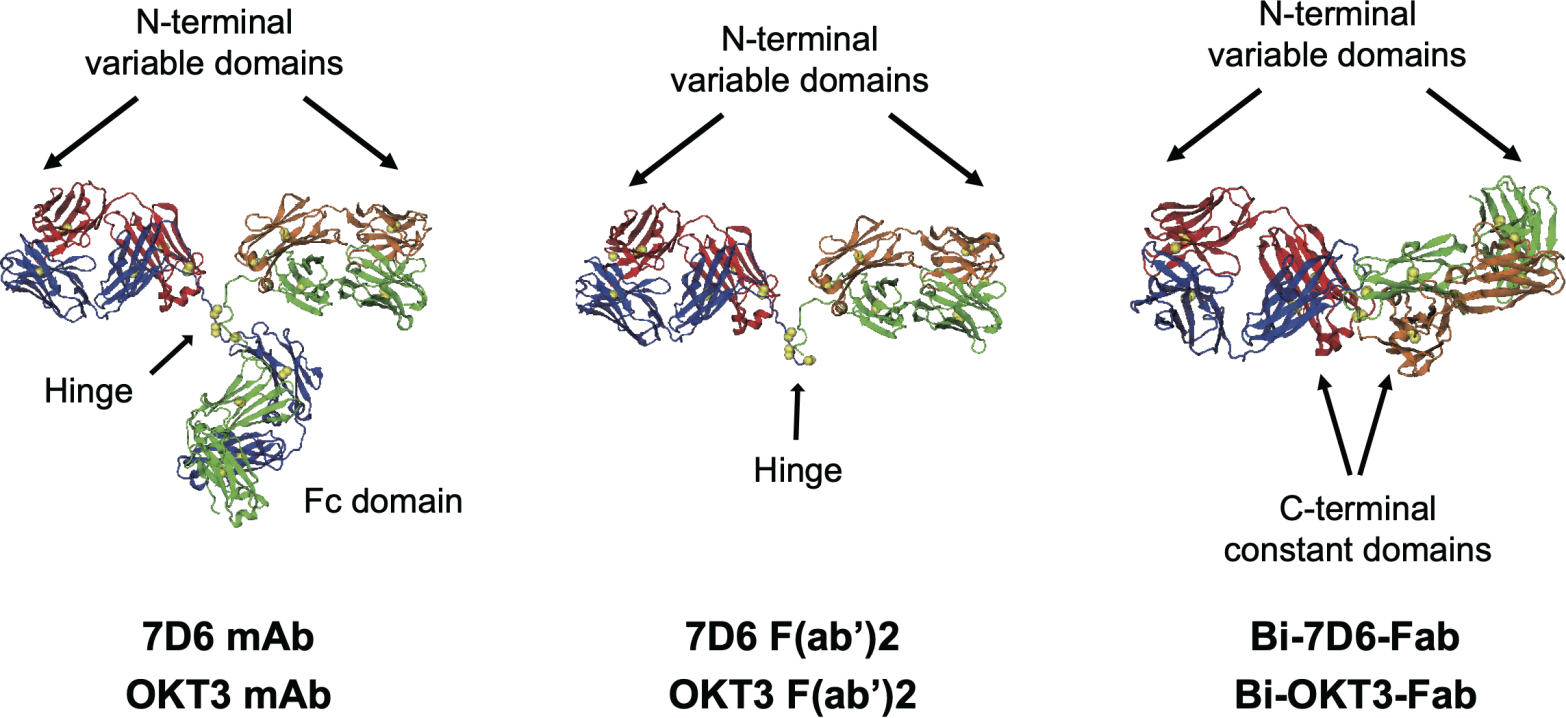
Structures of antibodies used in this work. Heavy chains are blue and green, light chains red and orange, disulfide sulfurs are yellow spheres. OKT3 F(ab’)2 and Bi-OKT3-Fab models shown were used for MD. The mAb and F(ab’)2 structures come from PDB 1IGT (33). The Bi-OKT3-Fab structure was derived in this work. Except for one constant domain difference, sequences of 7D6 and OKT3 only varied in the CD3ε-binding variable domains.

Here we studied the functional responses of T cells when stimulated with matching-specificity bivalent variants of anti-CD3ε: intact monoclonal antibodies (mAb), F(ab’)2 fragments and non-covalent dimers of Fab fragments (Bi-Fabs) (**Figure 1**). Bi-Fabs spontaneously accumulate as a minority species when preparing Fab fragments from mAb IgGs (16). Bi-Fabs can be purified by size exclusion chromatography and prevented from further aggregation when stored in the presence of high concentration of osmolytes (16). When ranked according to their capacity to stimulate cell division and cytotoxicity, anti-CD3 Bi-Fabs displayed the highest capacity to activate T cells over F(ab’)2 and mAb of the same specificity. To understand this unexpected outcome, we evaluated several structural features of Bi-Fab, F(ab’)2, and mAb as ligand free and ligand bound molecules using a combination of empirical and molecular modeling approaches. As ligand free moieties, Bi-Fabs presented reduced flexibility of anti-CD3 Fab domains to rotate with respect to each other, with these Fabs displaying angles closer to 180° than in F(ab’)2 and mAb molecules. Molecular dynamic simulations were performed to compare crosslinking of CD3 at the plasma membrane by Bi-Fab and F(ab’)2. Fabs from Bi-Fab and F(ab’)2 presented close to orthogonal angles between the Fab/epitope complex and the plasma membrane and engaged their epitopes in CD3 molecules anchored at opposing plasma membrane domains. Yet a greater range of angles between the Fab/CD3 epitope and the plasma membrane domains in anti-CD3 F(ab’)2 was observed than in the case of anti-CD3 Bi-Fab, suggesting the latter anti-CD3 crosslinker holds two opposing CD3 epitopes in a more rigid/less flexible configuration. We propose that lesser flexibility when bridging TCR/CD3 receptors located in opposing membrane domains explains the superior capacity to trigger T cell division and cytotoxicity displayed by the anti-CD3 Bi-Fabs.

Our findings suggest a potential path to optimizing the capacity of anti-CD3 BiAbs to bridge T cells to targeted cells, as well as to increase T cell cytotoxicity against such targets that could be followed to enhance the therapeutic performance of anti-CD3 BiAbs in patients.

## Materials and methods

### Mice

C57BL/6 (B6) and 129/SvImJ mice were purchased from The Jackson Laboratory. CD3ε−/− ζ−/− mice (T cell–deficient mice lacking all four CD3 subunits CD3γδεζ) (17) were originally provided by D. Vignali (St. Jude Children’s Research Hospital) with permission from Cox Terhorst (Beth Israel Deaconess Medical Center, Harvard Medical School). Perforin knockout mice were kindly provided by Aaron Johnson (Mayo Clinic, Rochester, MN, USA). All mice were used between 6-16 weeks of age. Mouse procedures were approved by Mayo Institutional or University of Missouri Animal Care and Use Committees and are consistent with National Institutes of Health guidelines for the care and use of animals.

### Antibodies and other reagents

The following panel of anti-TCR/CD3 monoclonal antibodies (mAbs) were purified from hybridoma supernatants: anti-mouse (Ms) CD3εγ (7D6 Ms IgG2a), anti-Ms CD3ε (145-2C11, hamster (Ham) IgG1), and anti-human CD3ε (OKT3 Ms IgG2a). The 7D6 hybridoma was kindly provided by Balbino Alarcón (Centro de Biología Molecular Severo Ochoa, Universidad Autónoma de Madrid, Spain). The 2C11 and OKT3 hybridomas were kindly provided by Ed Palmer (University Hospital-Basel, Switzerland). Abs from eBiosciences specific for mouse included anti-NK1.1, anti-B220, anti-CD11b, anti-CD11c, anti-MHC class II (2G9), anti-GR1, anti-Ter119, anti-Vβ5 (MR9-4), anti-Thy1.2 (53-2.1), anti-CD4 (RM4-5), anti-CD8α (53.6.7), anti-CD69 (H12F3), anti-CD25 (3C7), anti-Fas (15A7), and anti-CD19 (6D5). Annexin-V-PE was purchased from BD biosciences. Abs from Jackson ImmunoResearch included non-specific Ms, rat, ham IgG controls, human IgG mAb, and donkey anti-Ms IgG, goat anti-rat IgG, and goat anti-Ham IgG secondary Abs (raised against Heavy + Light chain immunogens) coupled to horseradish peroxidase (HRPO) for Western blots or coupled to FITC for flow cytometry. CpG oligodeoxynucleotide (CpG) (TriLink BioTechnologies) was synthesized with phosphorothioate backbone and HPLC purified with the following sequence, TCCATGACGTTCCTGACGTT. CTLA-4 Ig was purchased from BD Pharmingen, recombinant mouse Fas Fc chimera from R&D systems, and 96-well HTS 0.4 µm transwell plates from Corning.

### Preparation of Bi-Fab and F(ab’)2 fragments

Briefly, intact IgGs were digested with papain and Bi-Fabs were purified from monovalent Fab fragments (Mono-Fabs) by size-exclusion chromatography equilibrated in PBS as described previously (16). All Ig preparations were stored in sterile conditions at 4°C, at 0.2 mg/ml in PBS F(ab’)2 fragments were prepared by pepsin digestion (90 min, 37°C) of mAb using F(ab’)2 preparation kit (Thermo Scientific Pierce) according to manufacturer’s instructions.

### Peripheral lymphocyte isolation

Single cell suspensions from mouse splenocytes and lymph nodes were subjected to hypotonic shock to lyse red blood cells. In some experiments, resulting samples were then labeled with carboxyfluorescein succinimidyl ester (CFSE) prior culture and stimulation with control or specific IgGs. Purification of CD8 or CD4 T cells from peripheral lymphocytes was done by negative selection with a purity of >90% for CD4 or CD8 T cells. Briefly, negative selection was performed by incubating peripheral lymphocyte at 4-8°C in PBS supplemented with 2% Cosmic Calf serum (Hyclone) for 20 minutes with biotinylated Abs specific for NK1.1, B220, CD11b, CD11c, MHC class II (2G9), GR1, Ter119, and CD8a or CD4 for CD4 or CD8 purification, respectively. Cells were washed and incubated at 4-8°C with Miltenyi streptavidin microbeads (Miltenyi Biotec, Auburn, CA) for 15 minutes. Negative selection was carried out according to the manufacturer’s directions.

### *In vitro* generation of T cell blasts

Anti-CD3ϵ mAbs (2C11 (anti-mouse CD3ϵ) or OKT3 (anti-human CD3ϵ)) were bound to plastic wells at 10 µg/ml in PBS at 37°C for 1.5 hours. Wells were washed 3x with PBS to then be plated with samples of peripheral lymphocytes from murine spleens or peripheral mononuclear blood cells from healthy de-identified human donors. Plated samples containing 2.5 x 10^6^ total live cells per well in RPMI were supplemented with 10 U/ml of IL-2. Samples were cultured at 37°C for three days. On day three, cultures were transferred to175 cm^2^ flasks containing RPMI + 10 U/ml of IL-2. T cell blasts were used between day 5 and day 9 of starting the cultures.

### Binding assays of IgG mAb, F(ab’)2 and Bi-Fab to CD3ϵ

Triplicate samples of mouse peripheral lymphocytes (0.25x10^6^ viable cells/mL) were pre-incubated on ice for 60 minutes with Bi-Fabs specific for CD3ϵ diluted at 1.25 µg/ml, and F(ab’2) and mAbs specific for CD3ϵ or control IgGs diluted at 5 μg/ml. Next, samples were washed to be incubated on ice for 30 minutes with secondary anti-Ms or anti-Ham IgG-FitC. Then samples were washed to be incubated next on ice for 30 minutes with fluorescently labeled mAbs specific for Thy1.2, CD4, CD8α. All samples were washed after this last staining step and fixed in 0.25% formaldehyde prior to performance of flow cytometry using a C6 flow cytometer (Accuri Cytometers, Becton Dickinson). Data were analyzed using C6 Plus software or FlowJo v10.9 (BD Life Science) to observe binding of the different IgGs tested on live Thy1.2+ CD8+/CD4+ T cells.

### T cell stimulation with anti-CD3 IgGs

Whole cell suspensions from mouse spleen and lymph nodes were cultured in triplicate wells each containing 2.5x10^6^ viable cells T cells in 200 μl RPMI (Gibco) supplemented with 10% Cosmic Calf serum, for tissue culture at 10% CO_2_, 37°C. Anti-CD3 Bi-Fabs were tested for T cell stimulation at 1.25 μg/mL, while anti-CD3 mAbs and F(ab’)2 were used at 5 μg/ml. At indicated time points, plates were placed on ice and samples were stained for 30 minutes with fluorescently labeled mAbs specific for Thy1.2, CD4, CD8α, and different activation markers (24h-CD69, 48h-CD25, 72h-Fas (CD95)). After staining, samples were washed and subjected to flow cytometry using C6 flow cytometer. Data were analyzed using C6 Plus software or FlowJo v10.9 to observe up-regulation of cell surface expression of markers of T cell activation on live Thy1.2+ CD4+CD8**-** and Thy1.2+ CD4**-** CD8+ T cells. CD69 and CD25 were reported as % of CD4 or CD8 T cells positive over control treated samples. Fas was reported as median Fas fluorescence intensity found in CD4 or CD8 T cells. For counting viable T cells, cultures were stained after 96 h with fluorescently labeled mAbs specific for Thy1.2, CD19 and CD4, unless otherwise stated. When counting live re-stimulated blasted T cells, 5.0 x 10^4^ CD4 or CD8 blasts were plated in 96-well plates for at least 15 h at 37°C in 200 μl of RPMI media supplemented with the indicated anti-CD3 treatments. Next, cultures were washed and stained with fluorescently labeled mAbs specific for Thy1.2, CD4, and CD8α. Prior to FACS analysis using C6 Accuri flow cytometer, all samples were diluted into a final volume of 200 μl of FACS collection buffer. Propidium iodide (PI) was added at room temperature prior data collection of every single sample. A fixed volume of 150 μl was acquired for all samples. Live T cells were gated using exclusion of PI+ dead cells. Concentration of live T cells in the samples was obtained as counts of Live T cells/µL using C6 Plus software. Total T cell counts in samples were calculate by multiplying live T cell concentration data by 200 (final volume of samples prior data collection).

### Annexin-V staining

Triplicates 1.0 x 10^5^ total T cell blasts were cultured with the indicated soluble anti-CD3 IgG treatments in 96-well plates. At indicated time points cells were washed and stained with fluorescently labeled mAbs specific for CD4, and CD8α on ice for 30 minutes. Next, cells were washed and stained with 1:20 Annexin-V for 15 minutes at room temperature in 2.5 mM CaCl_2_, 140 mM NaCl, 10 mM HEPES, and pH 7.4 following manufacturer’s directions. PI was added at room temperature to every single sample prior flow cytometry using C6 flow cytometer. Data were analyzed using C6 Plus software to determine the frequency of CD4+ or CD8+ T cell blasts PI- Annexin V+ found in the samples.

### Experimental autoimmune encephalomyelitis

On day 0 male B6 mice received sub-cutaneous injections with 100 µg of the MOG peptide (aa 35-55) emulsified in CFA containing M. tuberculosis H37Ra (400 µg/mouse) in both flanks. On days 0 and 2 mice were injected intraperitoneally with Pertussis toxin (100 ng/mouse) (18, 19). From day 4 on, mice were periodically scored for external clinical symptoms of EAE as follows: 0 (no symptoms observed); 1 (loss of tail tone); 2 (hind limb weakness); 3 (hind limb paralysis); 4 (hind limb paralysis and forelimb paralysis or weakness); and 5 (moribund, mouse requiring euthanasia). Mice were terminated within two days when scored as 5.

### Statistical analyses

Statistics were performed using one-way ANOVA. Differences were significant at *p ≤*0.05. (ns p > 0.05, * p ≤ 0.05, ** p ≤ 0.01, *** p ≤ 0.001, **** p ≤ 0.0001) Data plotted in bar graphs reflect mean values +/**-** standard deviation (SD) or standard error (SE) as indicated.

### Electron Microscopy

Purified OKT3 and 7D6 Bi-Fab and F(ab’)2 were prepared for conventional negative staining with 0.75% (wt/vol) uranyl formate (UF). Briefly, Ab stocks stored in PBS were diluted 50-100x using 0.1x PBS, 5 μL samples were then applied to a glow discharged carbon coated grids for 2.5 min at RT, and excess protein was blotted with filter paper. Samples were then washed briefly twice with distilled water, incubated with 0.75% (w/v) UF for 20 seconds, excess UF removed with filter paper, and grids were air dried prior to data collection. Images were collected with a JEOL JEM 1400 transmission electron microscope equipped with a LaB6 filament and operated at an acceleration voltage of 120 kV. Images were recorded by using low-dose procedures on an UltraScan 1000 2k × 2k CCD camera (Gatan) using a defocus of −1.5 μm and a nominal magnification of 20,000-25,000× with a pixel size of 5.226 Å on the specimen level. Automated particle picking (auto-picking) was performed using two-dimensional classes generated using RELION version 3.0 software (20), a total of 6942 Bi-OKT3-Fab, 8071 Bi-7D6-Fab, 11730 OKT3 F(ab’)2, and 11290 7D6 F(ab’)2 particles were auto-picked using a threshold cutoff of 1.0, a maximum standard deviation of noise 1.1, and a minimum interparticle distance of 200 Å.

### Small angle x-ray scattering

Synchrotron radiation X-ray scattering data for purified OKT3 mAb F(ab’)2 and Bi-Fab were collected at the SIBYLS beamline (21–23). SAXS data collection parameters are summarized in **Supplementary Table S1**. Size exclusion chromatography (SEC) used PBS buffer. SAXS data analysis used Primus, Gnom, and FoXS (24–26).

### SAXS data collection and analysis

Standard procedures for synchrotron radiation data collection were achieved using the SIBYLS High Throughput SAXS Mail-in system at the Advanced Light Source 12.3.1 beamline (Berkley, CA). Aliquots (30 μl) of fresh samples and sample buffer were stored at 4°C in a 96-well full skirt PCR microplate (Axygen Scientific, CA) and centrifuged at 20,000 x g for 10 minutes prior to data collection. The plate was set on a cooling deck set to 10°C for the duration of the experiment. Concentrations of the samples included 2.83, 5.66 and 8.06 mg/ml for OKT3 monoclonal antibody (mAb), 1.5 and 3.0 mg/ml for Bi-OKT3-Fab, and 3.26, 3.45 and 3.87 mg/mL for OKT3 F(ab’)2. All sample buffers consisted of PBS, 137 mM NaCl, 2.7 mM KCl, 10 mM Na_2_HPO_4_ and 1.8 mM KH_2_PO_4_, and pH 7.4. Glycerol was added to all samples at a final concentration of 2% to minimize radiation damage. Exposure was performed using a monochromatic X-ray beam set with 12 keV energy and 10^11^ photon sec^-1^ flux with a detector distance set at 1.48 meters, corresponding to a momentum transfer, q ( $q=\;\frac{4\pi sin\theta }{\lambda }$ ), range of 0.01 to 0.032 Å^-1^. Data were collected at intervals with exposure times of 0.5, 1.0, 2.0 and 4.0 seconds. Buffer subtraction was achieved using averaged data collected on buffer prior to and following sample exposure. Buffer subtracted data for the low-angle region at low concentration were scaled with data at the low-angle region of the high concentration then merged with data at the high-angle region at high concentration (**Supplementary Figure S3**). Data analysis was performed using the ATSAS 2.7.2 software package. PRIMUS was used for evaluating Guinier and Porod plots and estimating the excluded volume of the hydrated molecule (Porod Volume, V_p_). The molecular mass (MM) was approximated using MM = V_p_/1.6 kDa for globular proteins. The ATSAS automated feature, AUTORG, was used to determine the scattering intensity, I(0), at q=0 and radius of gyration (R_g_) using the Guinier approximation within the low-q range (q*R_g_ ≤ 1.3). AUTOGNOM was used to calculate the pairwise distance distribution function P(r) and radius of gyration (R_g_^GNOM^) (27). The values of R_g_ and I(0) determined by AUTORG and AUTOGNOM were compared for consistency and then used for *ab initio* shape modeling.

Low resolution *ab initio* models were generated using GASBOR set with the following parameters: 1/Å angular units, P1 symmetry, reciprocal space mode and dummy residue number corresponding to the number of amino acids within each sample; 1316 for OKT3 mAb, 864 for OKT3 Bi-Fab, and 888 for OKT3 F(ab’)2 (28). GASBOR generated *ab initio* models were selected for averaging by visual inspection of fit between the GASBOR generated scatter profiles and SAXS data as well as the GASBOR automated discrepancy evaluation, χ^2^, defined by:


$$\chi^{2}=\frac{1}{n-1}\sum_{j}{\left[\frac{I_{exp}\left(s_{j}\right)-cI_{calc}\left(s_{j}\right)}{\sigma \left(s_{j}\right)}\right]}^{2}$$

Where n is the number of experimental points, σ(s_j_) is the experimental error, c is a scaling coefficient and I_exp_(s_j_) and I_calc_(s_j_) are experimental and calculated intensities at specified points at the momentum transfer s_j_, respectively. The DAMAVER program suite was used to build an averaged models from the GASBOR generated *ab initio* model libraries using 20 models with the lowest Chi values. The most probable model was selected by assigning a normalized spatial discrepancy (NSD) value for each model within the library then compared pairwise (29). The model with the lowest NSD value was then used as a reference for aligning and averaging the volume of all models. A cutoff volume was assigned to filter low occupancy and loosely connected atoms to yield a compact form of the most probable model.

### Bi-OKT3-Fab model construction

Coordinates for anti-CD3ϵ Fab, OKT3, came from PDB 1SY6 (30). It has two chains: L Gln1-Cys213 and H Gln214-Arg432. The balanced scoring scheme of ClusPro (31) produced 15 potential OKT3 dimer Bi-Fab models, #0-14. Heavy and light chains for Fab1 are H and L, those chains for Fab2 are A and B. Each chain has two intra-chain disulfide bonds and there is one heavy to light chain (H-L or A-B) disulfide bond. Swiss-Model (32) made the OKT3 F(ab’)2 model using PDB 1SY6 for Fab and PDB 1IGT (33) for the IgG hinge, which extends heavy chains H and A and includes three cysteines that crosslink them.

### Molecular dynamics

Molecular dynamics (MD) used Amber tools for system construction (34), GPU-enabled pmemd.CUDA (35, 36) for MD using ff14SB force field (37), and CPPTRAJ, and ParmEd and VMD for analysis (34, 38). Explicit solvent models were solvated in TIP3P water in truncated octahedron boxes of at least 12 Å to box edge using sufficient NaCl to neutralize and bring the final concentration to about 4.2 mM, consistent with the ionic strength of EM buffer. Amber tools ParmEd confirmed all disulfide bonds in the topology files. MD preparation started with two phases of 1000 step steepest descent and conjugant gradient energy minimization, first with protein atoms restrained (500 kcal mol^-1^ Å^-2^) and second without restraints. MD started by heating to 300 K over 20 ps under constant volume with protein restrained followed by unrestrained constant 1 bar pressure and temperature dynamics (NPT). Temperature regulation used Langevin dynamics with a one ps^-1^ collision frequency (39). Pressure regulation used a two ps relaxation time. Nonbonded cutoff was 9 Å and particle mesh Ewald calculated electrostatics using periodic boundary conditions (40). The SHAKE algorithm constrained bonds to hydrogens to allow a 2 fs time step (41). Production MD of 105 ns followed a 100 ps equilibration.

### Model analysis

Linear interaction energy (LIE) analysis monitored fab interaction during MD using relationship E_LIE_=0.5 E_electrostatic_ + 0.18 E_vdW_ (42, 43). Steered molecular dynamics (SMD) (44) tested potential Fab entanglement by pulling 24 constant domain Cα atoms in each Fab apart by 50 Å over 5 ns in implicit solvent with a 100 kcal mol^-1^ Å^-2^ force constant (36). Implicit solvent MD used generalized Born solvation with α=1.0, β=0.8, and γ=4.85 (45). Models with Bi-OKT3-Fab dimers bound to two membrane-embedded TCR/CD3 complexes used the eight-subunit TCR/CD3 structure PDB 6JXR (46). The vector sum of the first and last transmembrane helix Cα coordinates defined a transmembrane vector. This vector was normal to a curved 100 x 100 Å 1-palmitoyl-2-oleoyl-sn-glycero-3-phosphocholine (POPC) membrane used to embed TCR/CD3. Root mean squared (RMS) fitting of 50 shared CD3ϵ Cα atom coordinates of TCR/CD3 (PDB 6JXR chain f or chain e) and CD3ϵγ-OKT3 (PDB 1SY6) attached OKT3 to the TCR/CD3 structure. Then Bi-OKT3-Fab models attached to TCR/CD3 by RMS fitting Fab constant domain (103 L chain and 108 H chain) Cα atom coordinates shared by a Bi-OKT3-Fab model and TCR/CD3-OKT3. All fittings had an RMSD of less than 1.8 Å.

## Results

### Anti-CD3 Bi-7D6-Fab is competent to activate T cells

**Figure 2:**
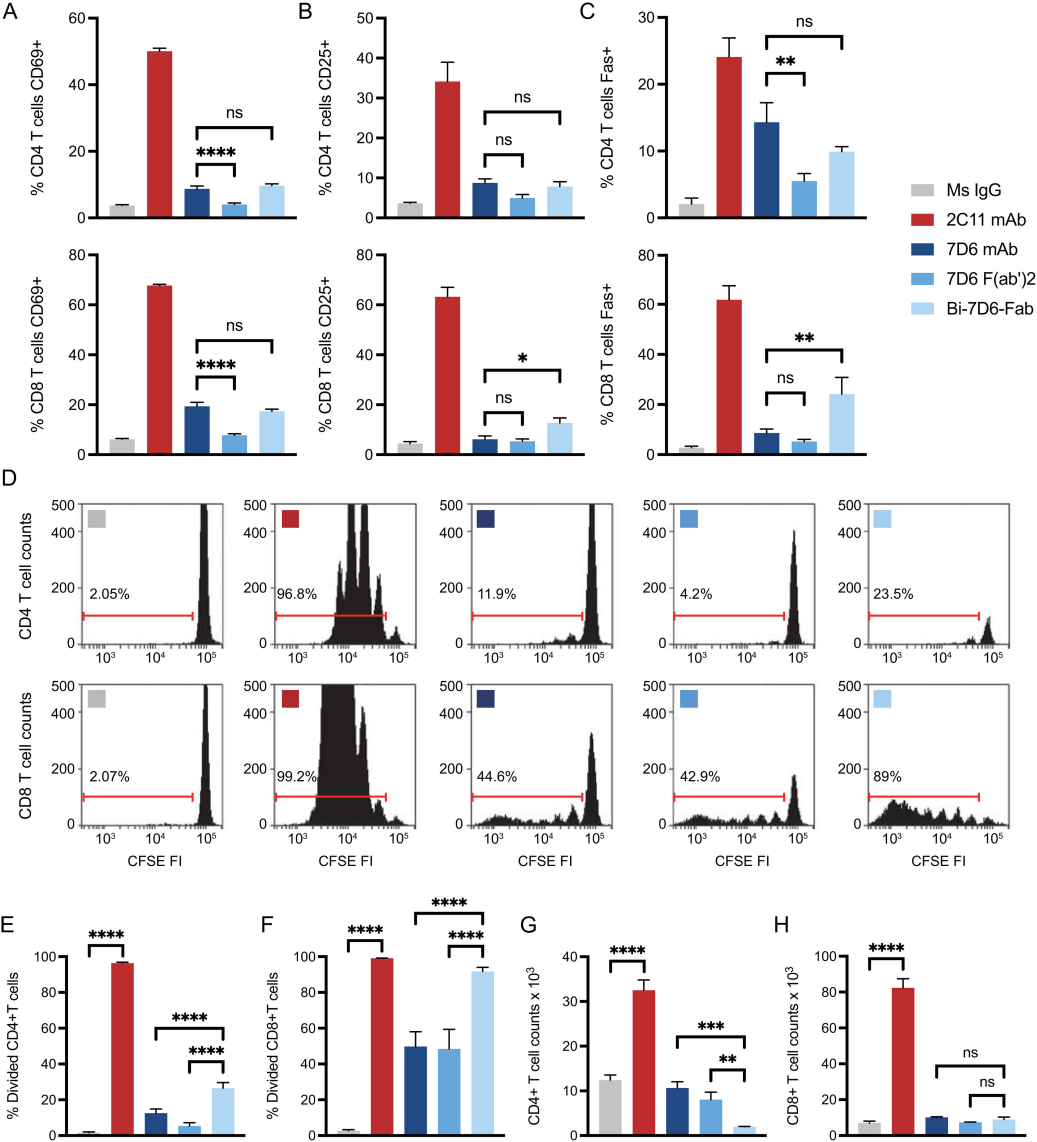
Anti-CD3 Bi-7D6-Fab is competent to activate T cells. **(A–D)** CFSE labelled B6 peripheral lymphocytes were incubated with CpG and soluble IgGs prior flow cytometry. Percentage of T cells positive for the expression of **(A)** CD69 at 24 h, **(B)** CD25 at 48 h and **(C)** Fas at 72 h over the levels found for each marker on gated T cells in unstimulated control Ms IgG samples. **(D)** CFSE dilution on gated T cells at 96 h. Ranged gates over plots indicate percentage of divided T cells according to CFSE dilution. **(E–H)** B6 peripheral lymphocytes labeled with CFSE were incubated with CpG and soluble IgG species for 96 h prior flow cytometry analysis. **(E, F)** Frequency (%) of CD4 **(E)** and CD8 **(F)** T cells dividing according to their CFSE profile. **(G, H)** Total counts of CD4 **(G)** and CD8 **(H)** live T cells. All samples were triplicated. Error bars represent +/-SD from replicas. One-way ANOVA test (ns p > 0.05, * p ≤ 0.05, ** p ≤ 0.01, *** p ≤ 0.001, **** p ≤ 0.0001).

Anti-mouse CD3ε Bi-7D6-Fab is a non-covalent dimer of Fab fragments of the mAb 7D6 (16). This antibody is specific for a conformational epitope found in the CD3ε chain when associated with the CD3γ chain of the CD3 complex associated with the T cell antigen receptor (TCR) (47). Bi-7D6-Fab triggers TCR/CD3 signaling based on robust upregulation of the surface marker CD69 (16). To establish its capacity to promote functional T cell responses, we compared Bi-7D6-Fab with 7D6 F(ab’)2 and 7D6 mAb in *in vitro* T cell stimulatory assays. Single cell suspensions of pooled spleen and lymph nodes from B6 mice were incubated with control mouse IgGs (MsIgG) or 7D6 mAb, 7D6 F(ab’)2, or Bi-7D6-Fab. As a positive control for proper T cell stimulation, cells were treated as well with anti-CD3ε mAb 2C11, which has superior capacity to induce T cell responses compared to 7D6 mAb (48) and has been used extensively to mimic agonist stimulation of murine T cells (49). All samples were treated additionally with the toll-like receptor agonist CpG to provide sufficient co-stimulatory signals in support of proper T cell activation (50, 51). As expected, 2C11 mAb treated samples presented the highest frequencies of T cells positive for activation markers monitored (**Figures 2A–C**). Meanwhile, 7D6 mAb produced a milder response according to observed frequencies of CD4 and CD8 T cells positive for activation markers tested (**Figures 2A–C**), in agreement with prior literature (48). Also expectedly, due to lacking the Fc portion, 7D6 F(ab’)2 was a weaker stimulatory agent of CD4 and CD8 T cell activation than 7D6 mAb (**Figures 2A–C**). However, samples stimulated with Bi-7D6-Fab presented frequencies of T cells positive for the monitored activation markers equivalent or higher than those found on samples treated with 7D6 mAb (**Figures 2A–C**), despite the Bi-Fab lacking an Fc portion as 7D6 F(ab’)2 does (**Figure 1**). When examining T cell division after 96 h of culture, 2C11 mAb treatment yielded the highest frequencies of divided T cells (**Figures 2D–F**), with cultures accumulating the highest counts of live T cells (**Figures 2G, H**). Strikingly, cultures with Bi-7D6-Fab contained higher frequency of divided CD8 and CD4 T cells than samples treated with 7D6 mAb or F(ab’)2 (**Figures 2D–F**). However, the number of total live CD8 T cells at the end point of the experiments were similar among the three 7D6 species (**Figure 2H**), while CD4 T cell numbers clearly contracted (**Figure 2G**) when treated with Bi-7D6-Fab. These observations showed that Bi-7D6-Fab is a competent stimulatory agent for CD4 and CD8 T cells that leads to their division surprisingly more efficiently than 7D6 mAb and F(ab’)2.

### Stimulation of T cells with Bi-7D6-Fab induces activation induced cell death (AICD) dependent on IL2 and Fas/FasL signaling

**Figure 3:**
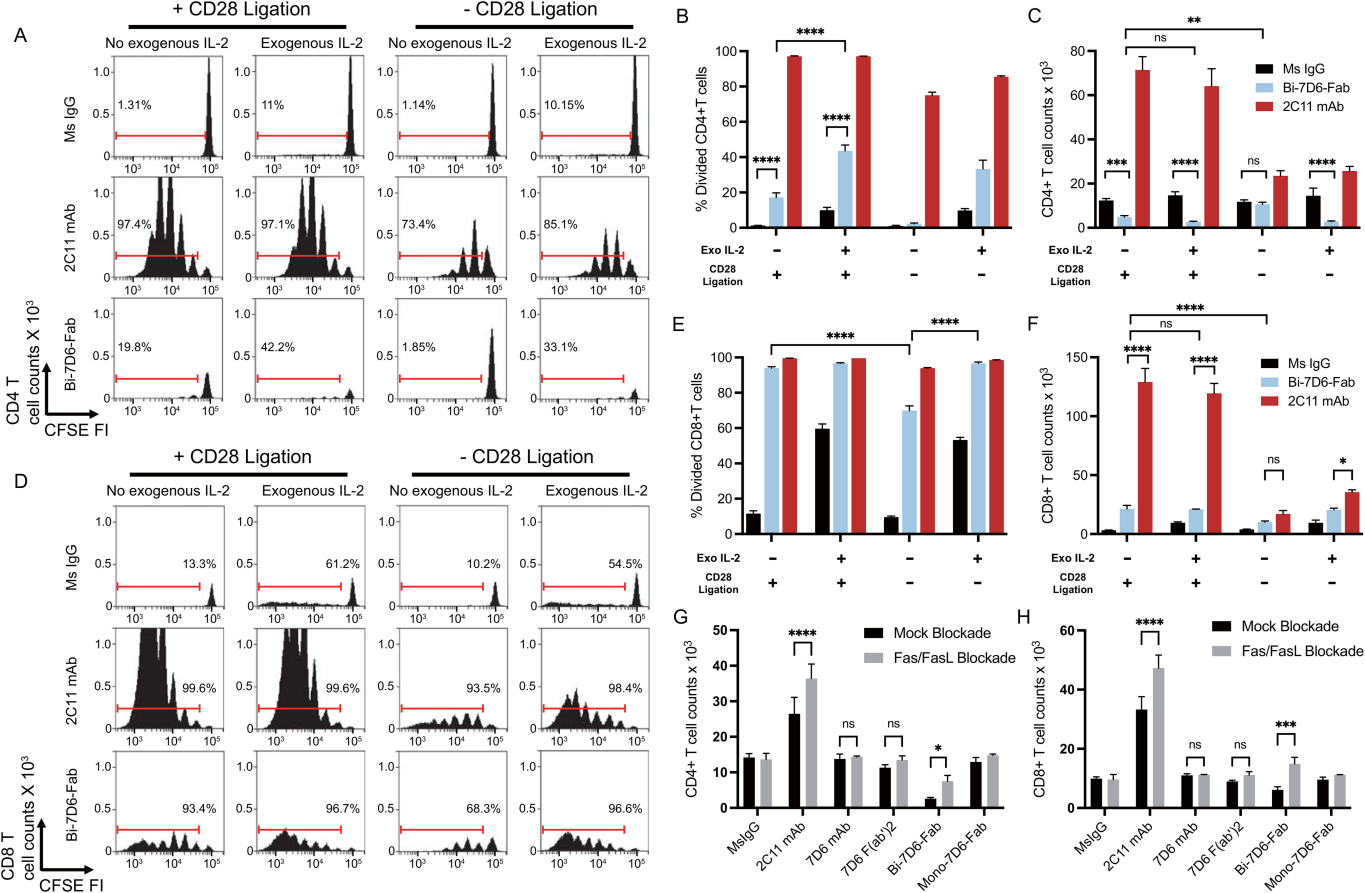
Stimulation of T cells with Bi-7D6-Fab induces AICD dependent on IL-2 and Fas/FasL signaling. **(A–F)** CFSE labeled B6 peripheral lymphocytes were incubated next with CpG and soluble IgGs in the presence of either Mock CD28 block (human control IgG) or CD28 block (human CTLA-4 IgG), and in the absence or presence of exogenous mouse IL-2. CFSE cell division profile, percentage division and total counts of live CD4 **(A–C)** and CD8 **(D–F)** T cells after 96 h. Red ranged gates in A and D indicate % of dividing T cells. **(G, H)** Next B6 peripheral lymphocytes were incubated with CpG and soluble IgGs in the presence of either Mock Fas/FasL block (control IgGs) or Fas/FasL block (Fas-Fc fusion protein and anti-FasL IgG) for 96 h. Total counts of live CD4 **(G)** and CD8 **(H)** T cells. Live CD4 T cells gated as PI- Thy1.2+ CD4+. Live CD8 T cells gated as PI- Thy1.2+ CD4-. All samples were triplicated. Error bars represent +/- SD from replicas when plotting frequency (%) values, and +/- SE from replicas when plotting counts. One-way ANOVA test (ns p > 0.05, * p ≤ 0.05, ** p ≤ 0.01, *** p ≤ 0.001, **** p ≤ 0.0001).

Since accumulation of live CD4 and CD8 T cells in cultures treated with Bi-7D6-Fab (**Figures 2G, H**) did not align with the frequencies of divided cells (**Figures 2D–F**), we next tested whether Bi-7D6-Fab might lead to the death of dividing T cells due to poor IL-2 production during the assays (52–54). Single cell suspensions of pooled splenocytes and lymph nodes from B6 mice were cultured with either control mouse IgG, 2C11 mAb or Bi-7D6-Fab, in the presence of either mock (+ CD28 ligation) or specific CD28 block (- CD28 ligation), and in the presence or absence of added IL-2. As shown prior in **Figure 2E**, after 96 h of *in vitro* culture, under + CD28 ligation and no IL-2 added condition, Bi-7D6-Fab stimulation resulted in only two rounds of detectable CD4 T cell division (**Figure 3A**) and a frequency of divided CD4 T cells below 20% (**Figure 3B**), while 2C11 mAb stimulation resulted in six detectable rounds of cell division (**Figure 3A**), and close to 100% divided CD4 T cells (**Figure 3B**). Despite detection of cells that had undergone division (**Figure 3A**), live CD4 T cell counts were significantly reduced in the presence of Bi-7D6-Fab when compared to unstimulated control treatment at 96 h (**Figure 3C**). Surprisingly, live CD4 T cell counts during stimulation with Bi-7D6-Fab were not significantly higher when IL-2 was added (**Figure 3C** + CD28 ligation), even though the frequency of divided CD4 T cells was increased by IL-2 addition. (**Figure 3B**, + CD28 ligation, - vs + IL**-**2). Conversely, when we used blocking agents of the CD28 signaling pathway to curtail endogenous IL**-**2 production during stimulation, Bi-7D6-Fab failed to reduce CD4 T cell counts when compared with unstimulated control (**Figure 3C**, **-** IL**-**2, **-** CD28 ligation), resulting in significantly higher CD4 T cell counts when comparing this condition with the Mock CD28 block control (**Figure 3C**, **-** IL**-**2, + vs – CD28 ligation). Finally, adding IL-2 to Bi-7D6-Fab stimulation in the presence of CD28 blockade re-established the observed reduction of CD4 T cell counts compared to the matching unstimulated control, resulting in significantly lower CD4 T cell counts than the combined treatment of Bi-7D6-Fab and CD28 block (**Figure 3C**, + IL**-**2, **-** CD28 ligation). These data indicated that IL-2 does not prevent but instead promotes unproductive cell division of CD4 T cells when single cell suspensions of peripheral lymphocytes are treated with Bi-7D6-Fab, suggesting the possibility of AICD as a mechanism of death for dividing CD4 T cells stimulated with Bi-7D6-Fab.

Next, we examined the cell division profile (**Figure 3D**), frequency of divided (**Figure 3E**) and live cell counts (**Figure 3F**) of CD8 T cells after 96 h of treatment in the same experimental samples described above. Under + CD28 ligation and no IL-2 added conditions, 2C11mAb and Bi-7D6-Fab treatments resulted into 6 detectable rounds of division of CD8 T cells (**Figure 3D**), inducing similar frequencies of divided CD8 T cells (**Figure 3E**). In contrast, live CD8 T cell counts in these cultures were significantly lower for Bi-7D6-Fab than 2C11 mAb (**Figure 3F**, + CD28 ligation, - IL-2). Addition of IL-2 to the cultures did not result in higher counts of live CD8 T cell upon Bi-7D6-Fab treatment (**Figure 3F**, + CD28 ligation). When comparing these results with cultures stimulated under blockade of the CD28 co-stimulatory pathway, cultures treated with Bi-7D6-Fab presented lower frequencies of divided CD8 T cells (**Figure 3E**, - IL-2, + vs – CD28 ligation) that increased with added IL-2 (**Figure 3E**, - CD28 ligation). Live CD8 T cell counts in Bi-7D6-Fab were significantly lower when CD28 ligation was blocked (**Figure 3F**, - IL-2, + vs – CD28 ligation). Thus, as seen for CD4 T cells, Bi-7D6-Fab induced CD8 T cell division as well as death dependent on endogenous IL-2 secretion, suggesting AICD as a mechanism of death for dividing CD8 T cells.

During AICD, T cells undergo apoptosis mediated by TCR/CD3 and Fas/FasL signaling pathways. Since Bi-7D6-Fab treatment increased frequencies of Fas positive CD4 and CD8 T cells (**Figure 2C**), we next examined if Bi-7D6-Fab-induced AICD required Fas/FasL signaling. *In vitro* cultures of single cell suspensions of peripheral lymphocytes from B6 mice were stimulated with either control mouse IgG or 7D6 mAb, 7D6 F(ab’)2, or Bi-7D6-Fab in the presence or absence of a Fas/FasL blockade regimen (55–57). As expected, stimulation with 2C11 mAb after 96 h resulted in higher accumulation of live CD4 and CD8 T cells in the presence of Fas/FasL blockade (**Figures 3G, H**). Accumulation of T cells due to Fas/FasL blockade were also observed in samples treated with Bi-7D6-Fab, but not for the weaker stimuli of 7D6 mAb and 7D6 F(ab’)2 (**Figures 3G, H**). Thus, stimulation of peripheral T cells *in vitro* with Bi-7D6-Fab induces AICD via Fas/FasL signaling in CD4 and CD8 T cells.

### Stimulation with Bi-7D6-Fab results in T cell fratricide

**Figure 4:**
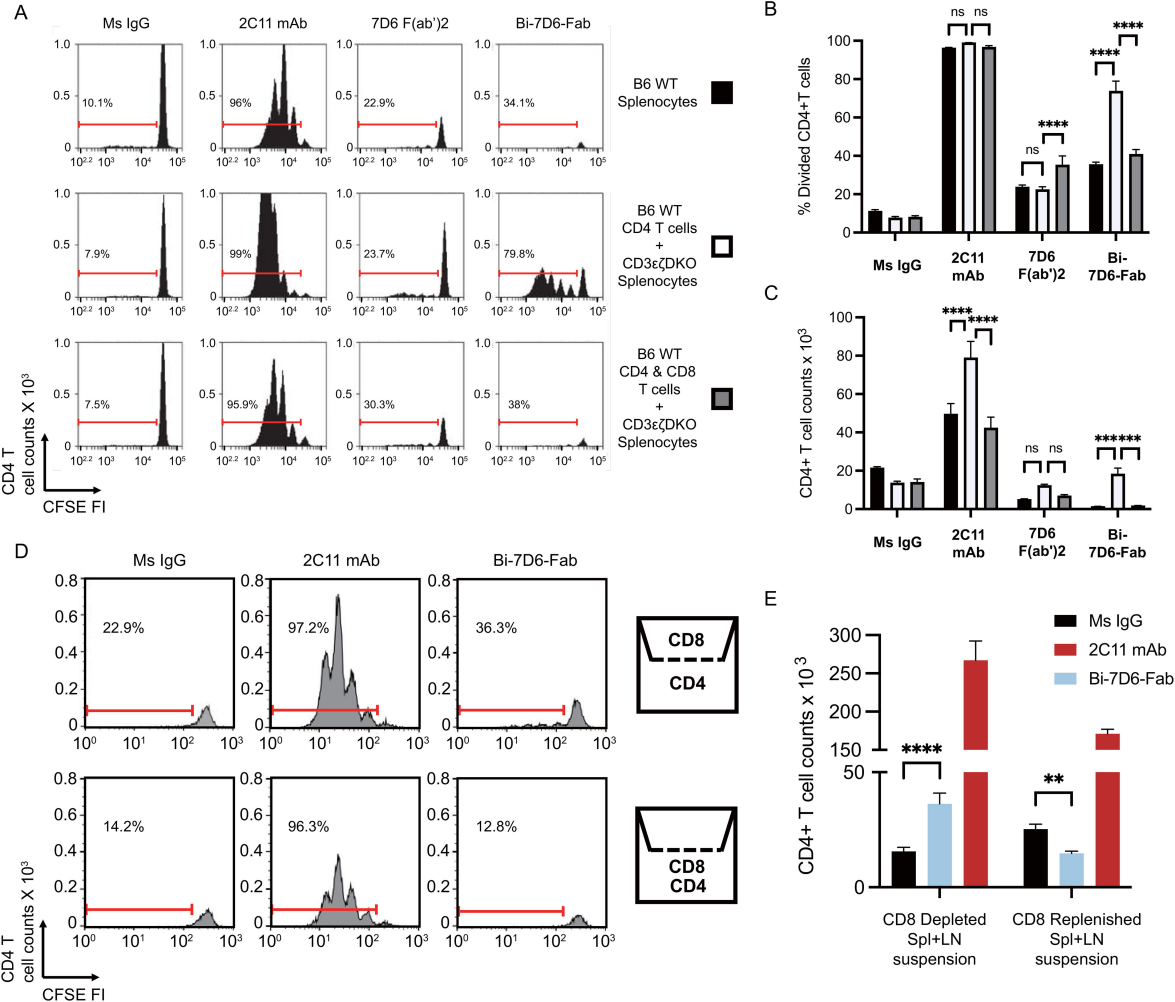
Crosslinking of the TCR/CD3 receptor by bivalent anti-CD3ε IgGs leads to T cell fratricide that involves cell to cell contact. **(A–C)** CFSE labeled B6 splenic lymphocytes were incubated with CpG and soluble IgGs for 96 h prior flow cytometry analysis. **(A)** CFSE cell division profiles with red ranged gates indicating frequencies (%) of dividing CD4 T cells. **(B)** Frequency (%) of CD4 T cells dividing according to their CFSE profile. **(C)** Total counts of live CD4 T cells. **(D, E)** Separate samples of purified splenic CD4 or CD8 T cells were CFSE labeled and then plated respectively into the lower and upper chambers of a trans-well plate or mixed into the lower chamber. Samples were then stimulated with CpG and soluble IgGs for 96 h prior flow cytometry analysis. **(D)** CFSE cell division profiles with red ranged gates indicating frequencies (%) of dividing CD4 T cells. **(E)** Total counts of live CD4 T cell counts. Live CD4 T cells gated as PI- Thy1.2+ CD4+. Divided CD4 T cells gated as shown in **(D)**. All samples were triplicated. Error bars represent +/- SE from replicas. One-way ANOVA test (ns p > 0.05, * p ≤ 0.05, ** p ≤ 0.01, *** p ≤ 0.001, **** p ≤ 0.0001).

Next, we hypothesized that T cells may engage in fratricide as part of their response to anti-CD3 crosslinking with the Bi-7D6-Fab. To test this hypothesis, we compared 7D6 Bi-Fab and 7D6 F(ab’)2 effects on the stimulation of CD4 T cells (potential fratricide targets) in the presence or absence of CD8 T cells (potential fratricide effectors). B6 splenocytes were used to purify CD4 or CD8 T cells while splenocytes from CD3ε^-/-^ζ^-/-^ double knock out mice lacking peripheral T cells were used as a source of APCs (17). In all, we cultured total B6 splenocytes, CD4 T cells with APCs, or CD4 plus CD8 T cells with APCs in the presence of control Ms IgG Fab, 7D6 F(ab’)2, Bi-7D6-Fab or 2C11 mAb. Total B6 splenocytes recapitulated prior experiments showing 7D6 Bi-Fab and F(ab’)2 induced greater CD4 T cell division (**Figures 4A, B**) but reduced live CD4 T cell counts (**Figure 4C**) compared with unstimulated controls. Meanwhile, 2C11 mAb induced much greater CD4 T cell division (**Figures 4A, B**) and increase of live cell counts than the rest of the stimuli applied (**Figure 4C**). However, the frequency of divided CD4 T cells treated with Bi-7D6-Fab was significantly higher in the absence of CD8 T cells than in the presence of CD8 T cells (**Figures 4A, B**), followed by a consequent higher accumulation of live CD4 T cells (**Figure 4C**). Such effects were not observed when purified CD8 T cells were replenished to the samples (**Figures 4A–C**). 7D6 F(ab’)2 stimulation did not result in elevated live CD4 T cell counts when CD8 T cells were absent compared to the total B6 splenocytes (**Figure 4C**), with no detectable changes in CD4 T cell division (**Figures 4A, B**). These results indicate that CD4 T cells exposed to 7D6 F(ab’)2 or Bi-7D6-Fab undergo cell division, but CD8 T cells engage in fratricide of CD4 T cells as part of their response to Bi-7D6-Fab stimulation. Interestingly, CD4 T cells also displayed higher live counts in the absence of CD8 T cells, significantly when 2C11 mAb stimulation was provided, and trending when 7D6 F(ab’)2 was the stimulus (**Figure 4C**). These observations suggest other bivalent IgG forms specific for CD3ε, such as 2C11 mAb and 7D6 F(ab’)2 also promote T cell fratricide.

### Bi-7D6-Fab driven fratricide requires T cell to T cell contact

Next, we speculated that fratricide required T cell to T cell contact. To determine whether this was the case, we performed an experiment with purified CD4, and CD8 T cell populations plated respectively into the lower and upper chambers of a trans-well plate to prevent admixing (**Figure 4D**). Similar to when CD8 T cells were absent from the stimulation assays in **Figures 4A, B**, the frequency of divided CD4 T cells found in the Bi-7D6-Fab treated condition was higher when CD8 T cells were isolated in the upper chamber than when they were admixed with CD4 T cells in the lower chamber (**Figure 4D**). Live CD4 T cell count in the Bi-7D6-Fab treated condition was significantly higher than Ms IgG control group when CD8 T cells were isolated in the upper chamber but reduced when CD4 and CD8 cells were admixed. (**Figure 4E**). These results indicate that direct cell to cell contact facilitates the observed fratricide of activated T cells via bivalent anti-CD3 antibodies. Together with cell division, this fratricide appears part of the response of T cells to activation because it requires IL-2 and Fas/FasL signaling as shown in **Figure 3**.

### Bivalent anti-CD3 IgG species directly bridge T cells to T cells during their fratricide

**Figure 5:**
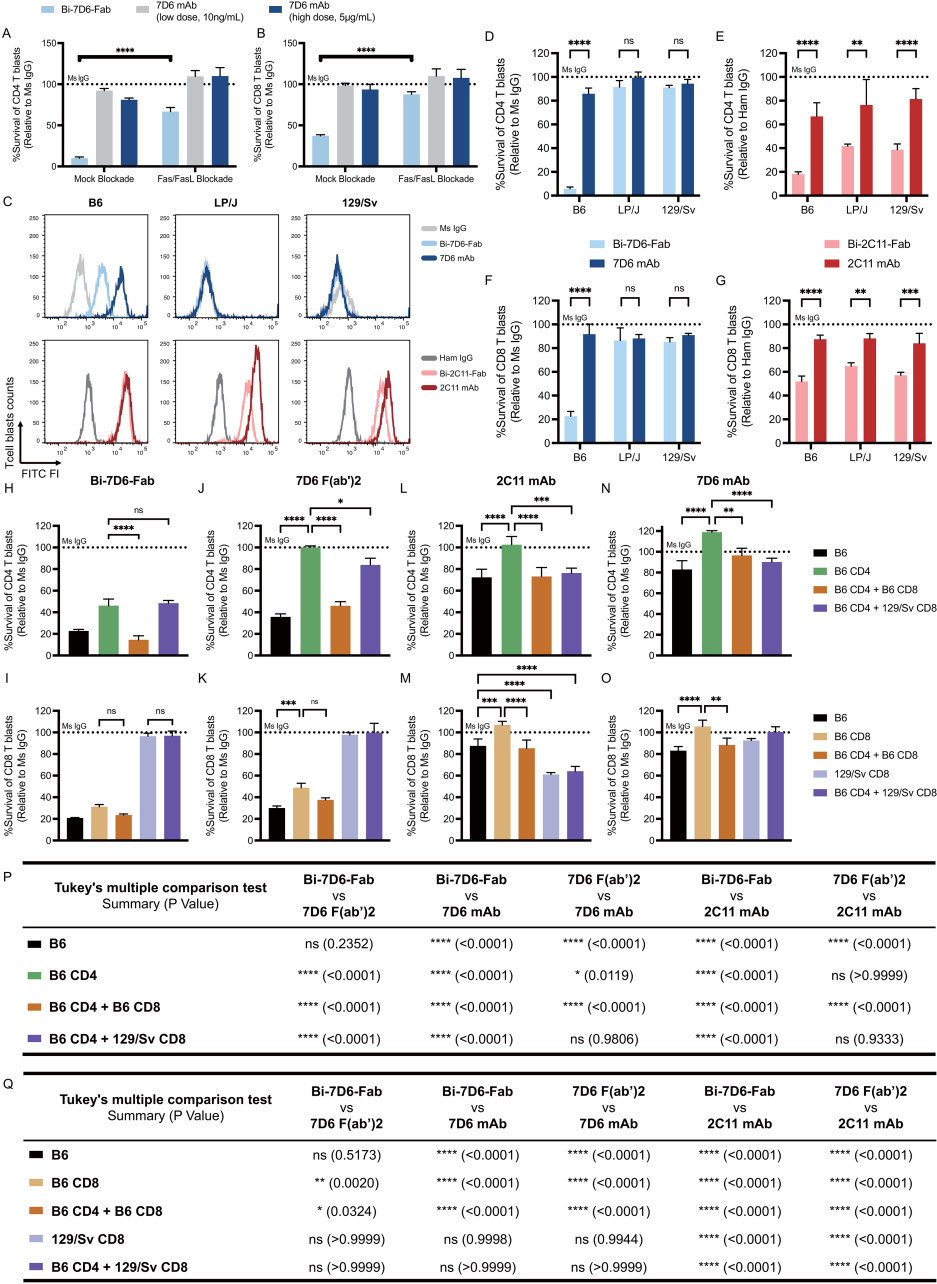
Bivalent anti-CD3 IgG species directly bridge T cells to T cells during fratricide. **(A, B)** B6 T cell blasts were incubated 24 h with mouse IL-2 and soluble IgGs in the presence of mock treatment or Fas/FasL blockade treatment to be subjected to flow cytometry analysis of T cell survival. Survival of T cell blasts treated with Bi-7D6-Fab or 7D6 mAb are plotted as a % of the survival found in CD4 **(A)** and CD8 **(B)** T cell blasts treated with Ms IgG. **(C)** Cell surface CD3ε (MFI) detected on live T blasts (PI- Thy1.2+) from B6, LP/J or 129/Sv mouse strains when stained with the indicated IgGs. **(D–G)** Unfractionated T cell blasts from the mouse strains B6, LP/J or 129/Sv were incubated with the indicated IgGs for 24 h prior flow cytometry analysis of live T cells. Survival of CD4 **(D, E)** or CD8 **(F, G)** T cell blasts as % of blasts surviving when incubated with control IgGs. **(H–O)** Unfractionated, fractionated or mixtures of fractionated B6 or 129/Sv CD4 and CD8 T cell blasts were incubated with IgGs for 24 h prior flow cytometry analysis of live T cells. Survival of T cell blasts incubated with anti-CD3ε IgGs is plotted as a % of the blasts surviving when incubated with control Ms IgG. All samples were triplicated. Error bars represent +/- SD from replicas. One-way ANOVA test (ns p > 0.05, * p ≤ 0.05, ** p ≤ 0.01, *** p ≤ 0.001, **** p ≤ 0.0001). **(P, Q)** Statistic results of comparing survival of CD4 **(P)** or CD8 **(Q)** T cell blasts data in panel **(H–O)** between indicated IgGs. Tukey’s multiple comparison has been applied for Two-way ANOVA test (ns p > 0.05, * p ≤ 0.05, ** p ≤ 0.01, *** p ≤ 0.001, **** p ≤ 0.0001) under consideration of multiple variables.

As seen with primary T cells, CD4 and CD8 T cell blasts were sensitive to a 24 h treatment of 1.25 μg/mL dose of Bi-7D6-Fab that drastically reduced their survival when normalized to Ms IgG Fab controls (**Figures 5A, B**). This reduction in survival was not observed when T cell blasts were exposed to a 5 μg/mL dose of 7D6 mAb, or to a lower dose of 10 ng/mL of 7D6 mAb to control for the possibility that trace amounts of 7D6 mAb in Bi-Fab preparations were mediating such effects (**Figures 5A, B**, Mock blockade).

Since T cell blasts recapitulate the death observed in primary T cells treated with Bi-7D6-Fab, we used them to explore further the mechanism by which Bi-7D6-Fab promoted T cell fratricide. As this fratricide requires T cell to T cell contact (**Figures 4D, E**), and Bi-7D6-Fab single specificity is for the extra-cellular domain of CD3ε, we speculated anti-CD3 Bi-Fabs may act as molecular bridges to crosslink activated T cells that in turn commit fratricide via a dual apoptotic mechanism involving Fas/FasL and perforin/granzyme B.

To test whether anti-CD3 Bi-Fabs could bridge T cells, we took advantage of the discrimination of allelic variants of CD3ε by 2C11 and 7D6 antibodies. Whereas 2C11 binds to CD3ε on T cells of B6, LP/J and 129/Sv mice, 7D6 only binds to B6 T cells (47) (**Figure 5C**). Accordingly, both 2C11 and 7D6 Bi-Fabs reduced survival rate of B6 CD4 and CD8 blasts when normalized to Ms IgG Fab control conditions, while LP/J and 129/Sv blasts only reduced their survival rate when exposed to Bi-2C11-Fab but not Bi-7D6-Fab (**Figures 5D–G**). First, these results extend the observation of T cell fratricide to additional anti-CD3ε specificities, i.e. 2C11 and 7D6 across the mouse strains tested. Second, fratricide was not observed when Bi-7D6-Fab did not bind to CD3 in LP/J and 129/Sv blasts consistent with the hypothesis that it requires anti-CD3 Bi-Fabs to physically bridge T cells (**Figures 5D, F**). Interestingly, these experiments also revealed survival rates under a 100% for CD4 and CD8 T cell blasts cultured with intact 2C11 (B6, LP/J and 129/Sv), and 7D6 mAbs (B6 but not LP/J and 129/Sv), although these species curtailed survival to a lesser extent than the Bi-Fab species.

To further test the bridging effect of anti-CD3 Bi-Fabs leading to T cell fratricide, we co-cultured CD4 and CD8 T cell blasts from B6 and 129/Sv mouse strains in different combinations: B6 unfractionated blasts, purified B6 CD4 blasts, purified B6 CD8 blasts, purified B6 CD4 with purified B6 CD8 blasts, purified B6 CD4 blasts with purified 129/Sv CD8 blasts and purified 129/Sv CD8 blasts only. All cultures were treated with either control Ms IgG, 2C11 mAb, 7D6 mAb, 7D6 F(ab’)2 or Bi-7D6-Fab. We observed that survival rates of both B6 CD4 and B6 CD8 single cultures dramatically dropped when they were treated with Bi-7D6-Fab (**Figures 5H, I**, below dashed line), indicating fratricide among either isolated CD4 or CD8 T cells is possible. A decreased survival rate was observed when B6 CD4 blasts were treated with Bi-7D6-Fab in the presence of B6 CD8 blasts (**Figure 5H**), while the presence or absence of B6 CD4 T cell blasts altered B6 CD8 T cell blast survival insignificantly when exposed to Bi-7D6-Fab (**Figure 5I**). In contrast, the additional drop in B6 CD4 T cell blasts survival rate was not observed when mixing these blasts with 129/Sv CD8 blasts that are not bound by Bi-7D6-Fab (**Figure 5H**). Thus, while both CD4 and CD8 T cells are competent in mediating their own fratricide when exposed to anti-CD3 Bi-Fabs, CD8s may have an additional mechanism, less prevalent in CD4s, that add to the death of CD4 T cells when both populations are mixed. Additionally, the observation that 129/Sv CD8 blasts are (i) incompetent to undergo fratricide under Bi-7D6-Fab exposure (**Figure 5I**) and (ii) fail to enhance CD4 fratricide (**Figure 5H**), strongly support the hypothesis that anti-CD3 Bi-Fabs mediate T cell fratricide by crosslinking T cells to each other. Finally, in these mixing experiments, 2C11 and 7D6 mAbs, and 7D6 F(ab’)2, significantly compromised viability of CD4 and CD8 blasts depending on their capacity to bind to them (**Figures 5J–O**). 7D6 F(ab’)2 was a less potent T cell fratricide inducer than Bi-7D6-Fab for purified and mixed CD4 and CD8 T cell blasts (**Figures 5P, Q**), whereas 2C11 and 7D6 mAb were even less potent than 7D6 F(ab’)2 (**Figures 5P, Q**), in agreement with prior experiments showing Bi-7D6-Fab has higher capacity to induce cell division and death on naïve T cells than the 7D6 F(ab’)2 and 7D6 mAb species (**Figures 2**, **4A-C**).

### Fratricide of actively cycling T cell blasts involves perforin as well as Fas/FasL

**Figure 6:**
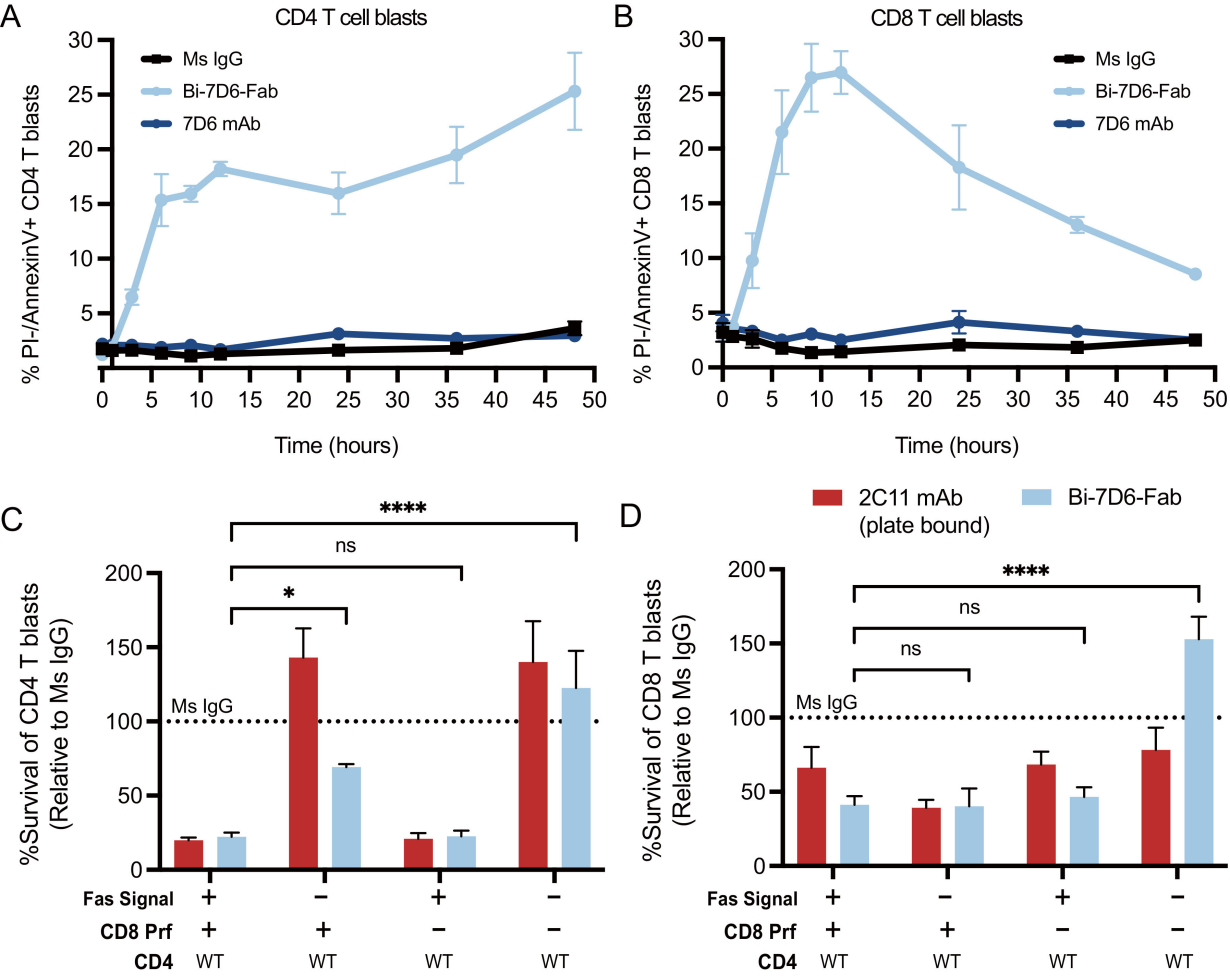
Actively cycling T cell blasts undergo apoptotic fratricide in response to Bi-7D6-Fab treatment mediated by Fas/FasL and Perforin. **(A, B)** B6 T cell blasts were incubated with mouse IL-2 and soluble IgGs and subjected to flow cytometry analysis at several time points to detect T cell apoptosis. % PI- Annexin-V+ CD4 (A) and CD8 **(B)** T cell blasts were plotted against time. **(C, D)** Fractionated CD4 and CD8 T cell blasts from either B6 WT or Prf1-/- mice were mixed and incubated with the indicated IgGs for 24 h in the presence of mock or anti-FasL and Fas-Fc blockade prior flow cytometry analysis of live T cells. Survival of CD4 **(C)** and CD8 **(D)** T cell blasts are plotted as a % of the blasts surviving when incubated with control IgGs. Live CD4 T blasts gated as PI- Thy1.2+CD4+CD8-. Live CD8 T cell blasts gated as PI- Thy1.2+ CD4-CD8+. All samples were triplicated. Error bars represent +/- SD from replicas. One-way ANOVA test. (ns p > 0.05, * p ≤ 0.05, ** p ≤ 0.01, *** p ≤ 0.001, **** p ≤ 0.0001).

The contraction of these cultures in the presence of Bi-7D6-Fab was dependent on the Fas/FasL signaling pathway since its blockade during Bi-7D6-Fab treatment significantly reduced the drop in CD4 and CD8 T cell survival rates observed after 18-20 h (**Figures 6A, B**, **-**/+ Fas/FasL blockade). CD4 and CD8 live T cell blasts turned positive for Annexin-V staining by 4 h of exposure to Bi-7D6-Fab, but not when exposed to 7D6 mAb, indicating T cells were undergoing apoptosis under exposure to Bi-7D6-Fab (**Figures 6A, B**).

Next, we tested whether cytotoxic function of CD8 blasts contributed to T cell fratricide. We prepared purified CD4 T cell blasts from B6 WT mice and co-cultured them with CD8 T cell blasts from either B6 WT or B6 knocked-out for the Perforin gene (Prf1^-/-^) (58). Co-cultures were performed in the presence or absence of a Fas/FasL blockade regimen and soluble Ms IgG Fab, soluble Bi-7D6-Fab or plate-bound 2C11 mAb. The later stimulus was added to provide a positive control for the apoptosis of T cell blasts caused by CD3 and Fas/FasL signaling leading to activation induced cell death (AICD) (59–61). As expected, in the presence of Fas/FasL signaling, co-cultures of WT CD4 with WT CD8 T cell blasts treated with Bi-7D6-Fab displayed reduced survival rate for both populations when normalized to Ms IgG Fab control, and close to the low survival rate found in the plate-bound 2C11 mAb condition (**Figures 6C, D**). When Fas/FasL signaling was blocked, survival of WT CD4 T cells increased to 60% of Ms IgG Fab control condition, while WT CD8 survival rate remained low (**Figures 6C, D**). On the one hand, these observations confirmed the contribution of Fas/FasL signaling in the fratricide of CD4 T cells, as well as revealed the added contribution of the cytolytic function of CD8 T cells. These observations also suggested that CD8 cytotoxic function can take over Fas/FasL mediated fratricide among CD8 T cells when the latter pathway is blocked. Surprisingly, survival rates of WT CD4 T cell blasts in the presence of Bi-7D6-Fab remained low rather than increase when co-cultured with Prf1^-/-^ CD8 T cell blasts. These observations suggested that Fas/FasL mediated apoptosis could predominate when perforin/granzyme B mechanisms were unavailable to mediate a fully effective fratricide of CD4 T cells. Finally, when the co-cultures of CD4 WT and CD8 Prf1^-/-^ were combined with the Fas/FasL blockade, the survival rates of CD4 and CD8 T cells elevated significantly over the rest of Bi-7D6-Fab conditions, to surpass survival rates in the Ms IgG Fab control condition (**Figures 6C, D**). These data, in combination with experiments isolating CD4 from CD8 T cells (**Figures 4D, E**) suggest a model for bi-valent anti-CD3 IgG mediated fratricide in which T cells, either CD4 or CD8, are efficiently killed by other T cells (CD4 or CD8) via Fas/FasL interactions, as well as by perforin/granzyme B mediated cytotoxicity when CTLs are involved.

### Potential therapeutic value of Bi-7D6-Fab depletion of T cells *in vivo*

Mounting evidence above showed Bi-7D6-Fab as a robust *in vitro* T cell stimulus leading activated T cells to kill and die in fratricide. Next, we investigated anti-CD3 Bi-Fabs efficacy to remove T cells *in vivo* (**Supplementary Figure S1**). B6 mice were injected intravenously (i.v.) with either 20 μg control Ms IgG Fab, Bi-7D6-Fab, 7D6 mAb (positive control for depletion of T cells by Fc effector mechanisms such as antibody dependent cellular cytotoxicity and complement dependent cytotoxicity) (ADCC), or 10 ng of 7D6 mAb (a control for trace presence of 7D6 mAb in Bi-7D6-Fab preparations). Mice were monitored periodically for piloerection and diarrhea during the following 24 h and bled to monitor for potential hypoglycemia and cytokine secretion. None of these tests provided evidence of cytokine release syndrome (CRS) (62, 63) induced by the IgGs injected (**Supplementary Table S2**). However, the ratio of T to B cell frequencies found in peripheral blood mono-nuclear cells (PBMCs) from mice injected with Bi-7D6-Fab dropped after 24 h compared to control Ms IgG Fab injected mice, although not to the extent observed when mice were treated with 20 μg of intact 7D6 mAb as a positive control for T cell depletion by ADCC and CDC (**Supplementary Figure S1A**). Next, B6 mice were injected i.v. three consecutive times, every 48 h, with either PBS or one dose of 20 μg of Bi-7D6-Fab. Mice were monitored up to two weeks for external signs of disease. The Bi-7D6-Fab injections induced a sustained reduction of the ratio of T to B cell frequencies in PBMCs during the two weeks compared with control mice (**Supplementary Figure S1B**), while all injected mice remained free of external signs of CRS. These data suggested that the injected Bi-7D6-Fab depleted T cells from peripheral blood without causing undiscriminated T cell cytokine secretion.

**Figure 7:**
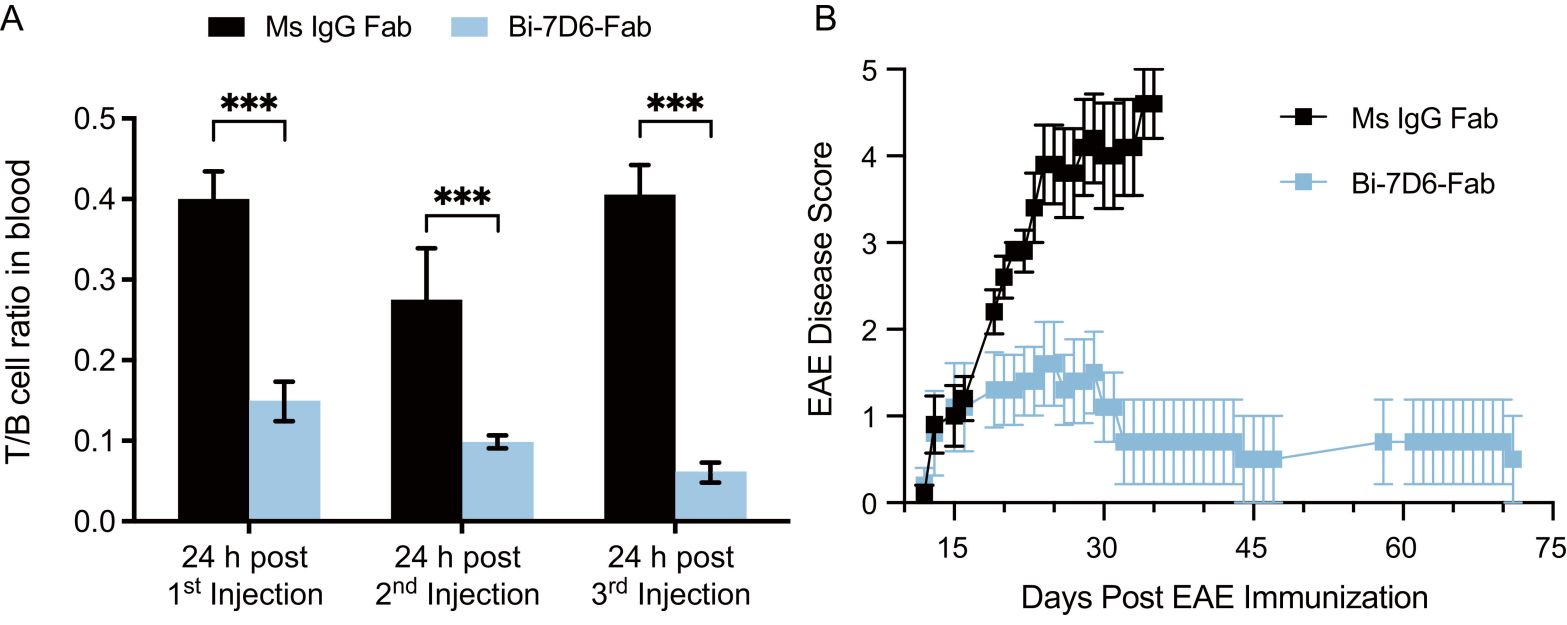
Bi-7D6-Fab depletion of peripheral T cells diminishes the course of EAE in B6 mice. B6 WT male mice were injected i.v. every 48 h from day 0 to 6 with a 20 μg dose of either Ms IgG Fab (n=5) or Bi-7D6-Fab (n=5). Three mice per IgG treatment were bled 24 h after each injection and PBMCs were isolated. **(A)** Ratio of T to B cell frequencies found in PBMCs by flow cytometry. Error bars represent +/- SE from replica samples. One-way ANOVA test (ns p > 0.05, * p ≤ 0.05, ** p ≤ 0.01, *** p ≤ 0.001, **** p ≤ 0.0001). On day 7, all mice were treated to induce EAE. Since then, mice were clinically scored for EAE external symptoms for 72 days when all surviving mice were terminated. **(B)** Average mean clinical score +/- SE of five mice per treatment was plotted against time.

Next, we injected B6 mice with the Ms IgG Fab versus Bi-7D6-Fab regimen described above. We observed again T cell depletion from peripheral blood after the first rounds of injections with Bi-7D6-Fab (**Figure 7A**). On day 6 after the course of Fab treatments, all mice were immunized with MOG_35-55_ peptide (emulsified with CFA, followed by administration of PTX at day 0 and 2 postimmunization) to induce experimental autoimmune encephalomyelitis (EAE), an animal model of multiple sclerosis (18, 19). Mice were scored blindly for the course of the experiment (**Figure 7B**). In the control group, mice treated with Ms IgG Fab displayed the expected progression of EAE disease with the disease onset around day 12, followed by ascending paralysis reaching a maximum score of 5, and a subsequent plateau starting from day 30. (64) (**Figure 7B**). Although treatment with Bi-7D6-Fab resulted in a comparable disease onset, mice receiving that treatment showed significantly reduced disease severity. Notably, the average maximum daily scores dropped in mice treated with Bi-7D6-Fab, beginning from day 16 post-immunization, remaining at a lowest value until all mice in that group were terminated on day 72 (**Figure 7B**).

In MOG_35-55_ induce EAE in B6 mice, a pathogenic autoimmune response orchestrated by CD4 T cells that specifically recognize the MOG_35-55_ peptide leads to central nervous system inflammation and demyelination (19, 65). Thus, the results described above indicate that despite lacking an Fc portion, Bi-7D6-Fab can efficiently deplete T cells *in vivo*, suggesting that T cells depleted may include subtypes actively engaged in a pathogenic process such as EAE. Our data also show Bi-7D6-Fab slowed EAE progression without causing damaging side effects related to overt stimulation of CD3 crosslinked T cells, which are common for other therapeutics based on anti-CD3 mAbs that have an Fc portion (66).

### Activated human T cells die by apoptosis when exposed to anti-human CD3ε Bi-OKT3-Fab

**Figure 8:**
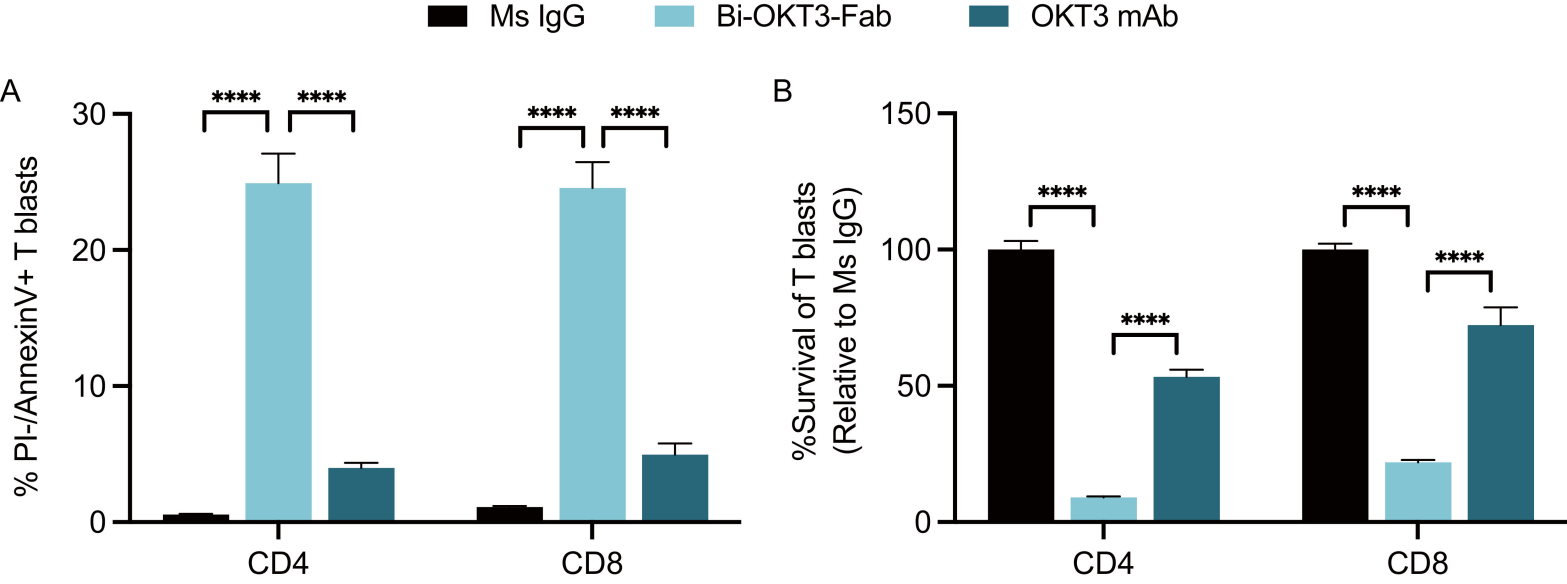
Activated human T cells die by apoptosis when exposed to anti-human CD3ε Bi-OKT3-Fab. Human T cell blasts were treated with soluble IgGs for 24 h prior flow cytometry analysis of apoptosis and survival. **(A)** % of live CD4 and CD8 T cell blasts undergoing apoptosis as PI-, Annexin V+. **(B)** Survival of CD4 and CD8 T cell blasts when treated with OKT3 mAb or Bi-Fab are plotted as % of T cell blasts surviving when treated with control Ms IgG. Live CD4 T cell blasts gated as PI- Thy1.2+ CD4+ CD8-. Live CD8 T cell blasts gated as PI- Thy1.2+ CD4- CD8+. All samples were triplicated. Error bars represent +/- SD from replicas. One-way ANOVA test. (ns p > 0.05, * p ≤ 0.05, ** p ≤ 0.01, *** p ≤ 0.001, **** p ≤ 0.0001).

We next studied the potential to drive T cell fratricide by Bi-OKT3-Fab, derived from a mouse IgG and specific for human CD3ε (67). Human CD4 and CD8 T cell blasts generated from healthy blood donors underwent robust apoptosis when exposed to Bi-OKT3-Fab, in contrast to their minimal response to OKT3 mAb (**Figure 8A**). Survival rates of these blasts cultured with Bi-OKT3-Fab were drastically reduced when normalized to equivalent cultures treated with the control Ms IgG Fab (**Figure 8B**). Although less robustly than Bi-OKT3-Fab, OKT3 mAb also reduced viability of cultured CD4 and CD8 human T cell blasts. These data align with observations made with Bi-7D6-Fab and 7D6 mAb (**Figure 6**), suggesting bivalent crosslinking of human CD3ϵ by OKT3 in Bi-Fab and mAb formats induces fratricide of human T cells as 7D6 and 2C11 do for murine T cells.

### Anti-CD3 7D6 and OKT3 Bi-Fabs adopt similar global shapes

**Figure 9:**
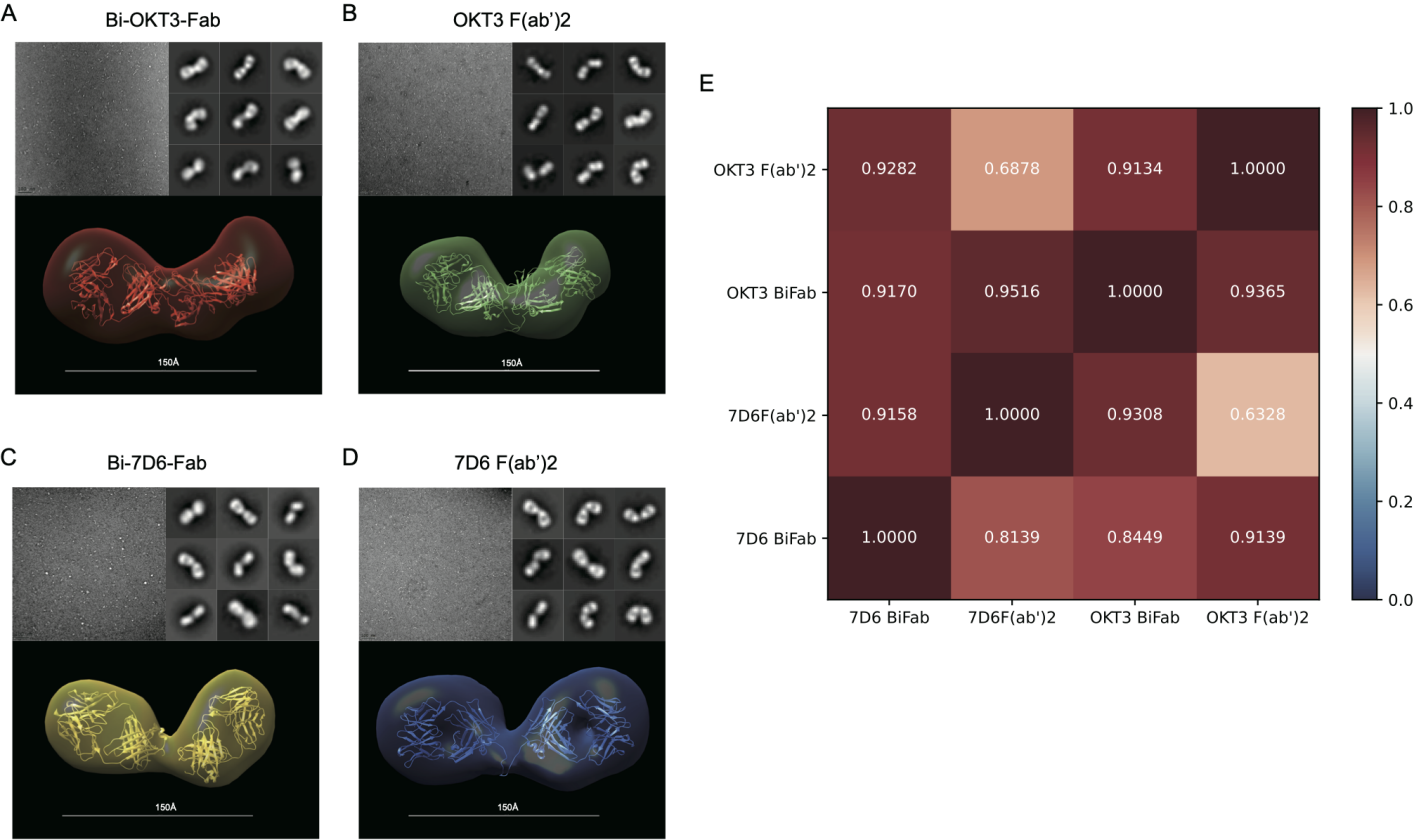
F(ab’)2 and Bi-Fab molecules have similar global shapes **(A–D)** Top-Left: Representative images for the optimized negative staining (OpNS) Electron Microscopy (EM) images of the indicated immunoglobulins. Top-Right: Examples of 2D-class averages for the indicated immunoglobulins for the 3D electron density maps. Bottom: Reconstructed 3D electron density maps overlaid with their corresponding homology models. **(E)** Goodness of fit between indicated immunoglobulins was calculated by Chimera with Fit in volume function based on spatial coincidence.

As shown above, anti-CD3 Bi-Fabs appear as a more potent stimulatory agent for T cells than F(ab’)2 of matching specificity. Differences in the response of T cells when exposed to either anti-CD3 F(ab’)2 or Bi-Fab could be due to structural differences among these molecular complexes. Using optimized negative staining electron microscopy (OpNs-EM) to compare 7D6 and OKT3 Bi-Fab and F(ab)’2, we found that (i) Bi-Fabs and F(ab’)_2_ antibodies have similar global shapes with anisometric extended bi-lobular structures (**Figure 9**) and (ii) Bi-Fabs from different clones (7D6 and OKT3) adopt similar shapes (**Figures 9A-D** bottom). Statistical evaluation of the structural data was performed by applying fitting cross-correlations (20) between the OKT3 and 7D6 Bi-Fab and F(ab’)2. The high correlation factor confirmed that all the molecular species share a similar bi-lobular structure (**Figure 9E**). Yet, using auto-picked 2D-classes (20) of the immunoglobulins (**Figures 9A-D**, top right), we observed Bi-Fabs (**Figures 9A, C**, top right) adopted a more linear conformation than their F(ab’)2 (**Figures 9B, D**, top right) in both clones 7D6 and OKT3.

### Analysis of OKT3 mAb, F(ab’)2 and Bi-Fab solution structures using SAXS

Next, we hypothesized that different flexibility between Fab moieties in mAb, F(ab’)2 and Bi-Fab antibodies could explain their different potency to activate T cells. In terms of structure, the presence of a hinge in the mAb and F(ab’)2 antibodies (**Figure 1**) could support a more flexible connection of the two Fabs attached than in the Bi-Fab complexes where a hinge is missing, and a more linear conformation is observed (**Figure 9**).

Since the structure of OKT3 Fab bound to CD3ϵ has been solved (PDB 1SY6) (30), we focused on this IgG and compared the conformational heterogeneity of OKT3 mAb, F(ab’)2 and Bi-Fab in solution to pursue any potential differences in Fab flexibility across these variants that could be associated with the different stimulatory responses observed on T cells. OKT3 and 7D6 Bi-Fabs display similar capacity to promote T cell fratricide (**Figures 5**, **8**), have similar shapes (**Figure 9**) and share nearly identical constant regions (**Supplementary Figure S2**). Thus, any findings from the comparison of the OKT3 structures (mAb, F(ab’)2 and Bi-Fab) could be applicable to the 7D6 variants.

**Figure 10:**
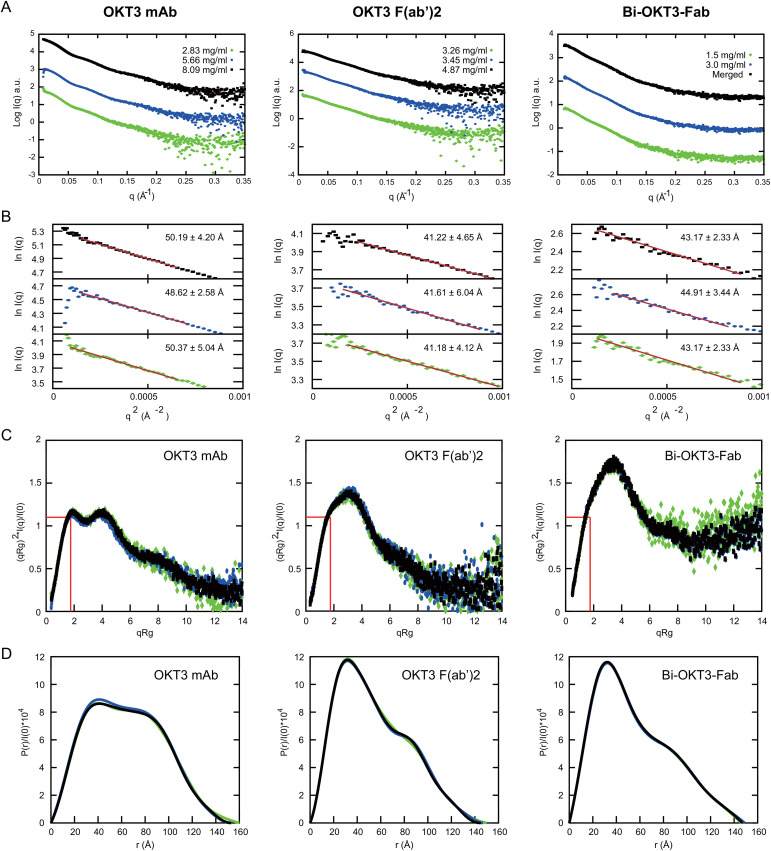
Small angle x-ray scattering (SAXS) analysis of OKT3 mAb, F(ab’)2 and Bi-OKT3-Fab **(A)** Scattering intensity plots (Log I(q) versus q) of indicated Igs. Bi-Fab merged scattering curve merges scattering data at low q range from the lower concentration with the higher q range from the higher concentration using Primus. **(B)** Guinier plots (Ln I(q) versus q2) corresponding to the SAXS curves in A. with the mAb (left), F(ab’)2 (center), and Bi-Fab (right). **(C)** Dimensionless Kratky plots with the position of Guinier–Kratky points ( $\sqrt{3}$ , 1.103) labeled with red lines, which is the main peak position for globular proteins (107). **(D)** normalized interatomic distance distribution (P(r)/I(0)) for the indicated OKT3 Igs.

We first studied solution conformation of OKT3 mAb, OKT3 F(ab’)2, and Bi-OKT3-Fab using small angle x-ray scattering (SAXS) at multiple concentrations (**Supplementary Table S3**, **Figure 10**). Analysis of the SAXS intensity curves and Guinier plots (68–70) indicated no aggregation over the concentrations studied (**Figures 10A, B**). The large q data had little influence on the calculated radius of gyration (R_g_) because biophysical parameters from Guinier and Porod plots (71) were consistent (**Supplementary Table S3**). We used SAXS data to determine molecular weights (MW) of the OKT3 species, which were acceptably close to the theoretical MW for OKT3 Igs (**Supplementary**-**Table S3**) calculated with the sequences for hinge regions and constant heavy regions 2-3 (CH2 and CH3) from murine IgG2 antibody sequence (PDB ID: 1IGT) (33). The normalized Kratky plot (I(q)_x_q^2^ versus q) (68–70) for OKT3 mAb was consistent with a globular protein with multiple domains that are regularly spaced (**Figure 10C**) (71). Kratky plots for the OKT3 F(ab’)2 and Bi-OKT3-Fab showed peaks shifted up and to the right of the Guinier-Kratky point (**Figure 10C**), which are consistent with the bi-lobular shapes seen by OpNs-EM in **Figure 9**. Since Kratky plots distinguish between extended and compact conformations (72), the distance distribution functions (**Figure 10D** and **Supplementary**-**Table S3**) of OKT3 mAb and F(ab’)2 indicate that at lower concentrations OKT3 Igs are in a more open and extended conformation with slightly larger maximum dimension (D_max_), than at higher concentrations when they adopt a slightly more compact and less extended conformation (**Figure 10D** and **Supplementary Table S3**). Bi-OKT3-Fab had a similar distance distribution profile to OKT3 F(ab’)2 but slightly more extended and with a less pronounced shoulder (**Figure 10D**). Additionally, Bi-Fab and F(ab’)2 measured D_max_ were similar, at ~147 Å for Bi-Fab compared to 145-150 Å for F(ab’)2 (**Supplementary Table S3**). In summary, Kratky plots of OKT3 Igs indicate that protein concentration has lower impact on the compactness in Bi-OKT3-Fab compared to OKT3 mAb or OKT3 F(ab’)2. According to the pattern of distance distribution in different OKT3 Igs, Bi-OKT3-Fab adopt slightly more extended conformation compared to OKT3 mAb or OKT3 F(ab’)2.

### OKT3 mAb and F(ab’)2 are flexible molecules that prefer conformations with Fab-Fab angles >100°

**Figure 11:**
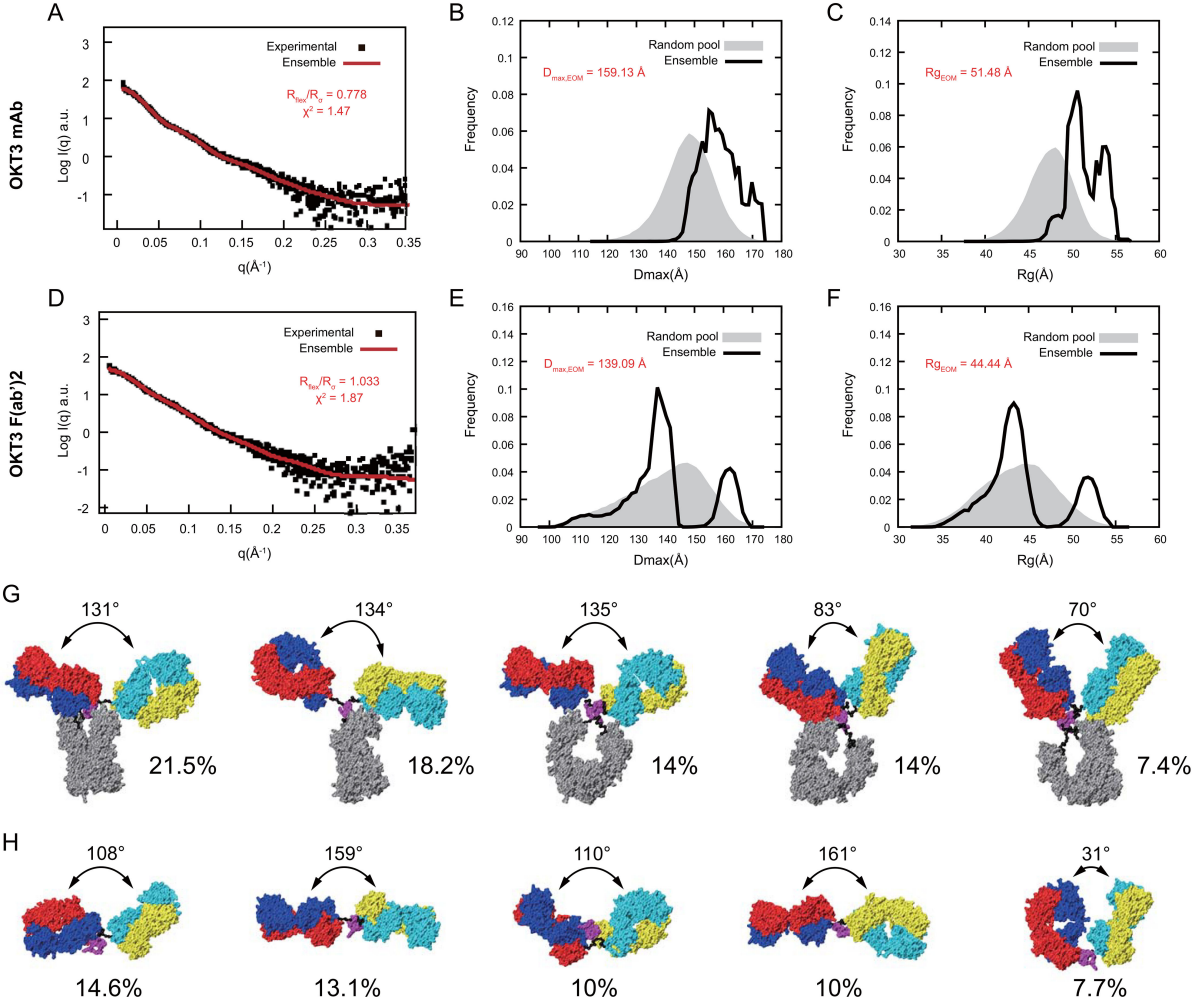
OKT3 mAb and F(ab’)2 are flexible and preferentially adopt more Fab-Fab conformations >100° Ensemble optimization for mAb and F(ab’)2 using experimental SAXS curves and homology models. Fitting of the optimized ensemble SAXS curve to the experimental **(A)** mAb and **(D)** F(ab’)2 SAXS curves. Overlay of optimized ensemble Dmax values for the random pool and selected ensembles for **(B)** mAb and **(E)** F(ab’)2. Overlay of optimized ensemble Rg values for the random pool and selected ensembles for **(C)** mAb and **(F)** F(ab’)2. Rflex: Metric for quantitative measure of flexibility. Rσ: Variance of the distributions of the selected ensemble and that of the pool. Top five conformers identified by the optimized ensemble method for **(G)** mAb and **(H)** F(ab’)2.

To quantitatively compare flexibility of OKT3 mAb and F(ab’)2 in solution, we applied the ensemble optimization method (EOM) (73). Using the OKT3 Fab sequence, and flexible linkers, hinge, and Fc domain of murine IgG2a antibody (PDB 1IGT; (33)), we obtained high quality fits (χ^2^<2.0) (74) between experimental SAXS data and an optimized ensemble with χ^2^ of 1.47 and 1.87 for mAb and F(ab’)2, respectively (**Figures 11A, D**). EOM metrics R_flex_ and R_σ_ quantitate flexibility and statistical variance of size distributions in constructed ensembles (73), high R_flex_/R_σ_ values indicate greater flexibility. Our EOM analysis shows that F(ab’)2 is more flexible than mAb with an R_flex_/R_σ_ of 1.033 and 0.778 respectively (**Figures 11A, D**), consistent with decreased steric hindrances in the absence of the Fc portion of the antibody. The D_max_ for mAb has a broad range from ~145-170 Å with two major R_g_ peaks around 50 and 53 Å (**Figures 11B, C**). The D_max_ for F(ab’)2 has three distinct peaks at ~111 Å, ~138 Å, and 160 Å and with two distinct R_g_ peaks at ~43 Å and ~51 Å (**Figures 11E, F**). The optimized ensemble for OKT3 mAb indicated that it prefers T shaped conformations with Fab-Fab angles of ~130° 72% of the time, and Y shaped conformations with Fab-Fab angles of<85° just under 29% of the time (**Figure 11G**, **Supplementary Table S4**). The optimized ensemble for OKT3 F(ab’)2 demonstrates that it prefers more linear T shaped conformations most of the time with Fab-Fab angles of ~110° and ~160° and V shaped conformations 14% of the time (**Figure 11H**, **Supplementary Table S4**).

### Identification of stably interacting Bi-OKT3-Fab conformers using molecular dynamic simulations

Due to the non-covalent nature of Fab association in Bi-Fabs, and unsatisfactory buffer subtraction in the Bi-OKT3-Fab SAXS sample (**Supplementary Figure S3**), EOM was not amenable to studying Bi-OKT3-Fab conformational heterogeneity, so we sought a method to generate a pool of stably interacting Bi-OKT3-Fab conformers to compare to the experimental SAXS data on Bi-OKT3-Fab. To generate a pool of stably interacting Bi-Fab conformers, we first used the docking program ClusPro to generate unbiased energetically favorable Bi-OKT3-Fab models using two copies of OKT3 Fab. In thirteen of the resulting fifteen models, Fabs interacted via the C-termini of their constant regions, leaving OKT3 variable domains free to bind CD3ϵ. Models 12 and 13 were not considered further because Fabs associated through variable-variable domain interactions, which precludes binding to CD3ϵ and is therefore inconsistent with our functional observations above.

**Figure 12:**
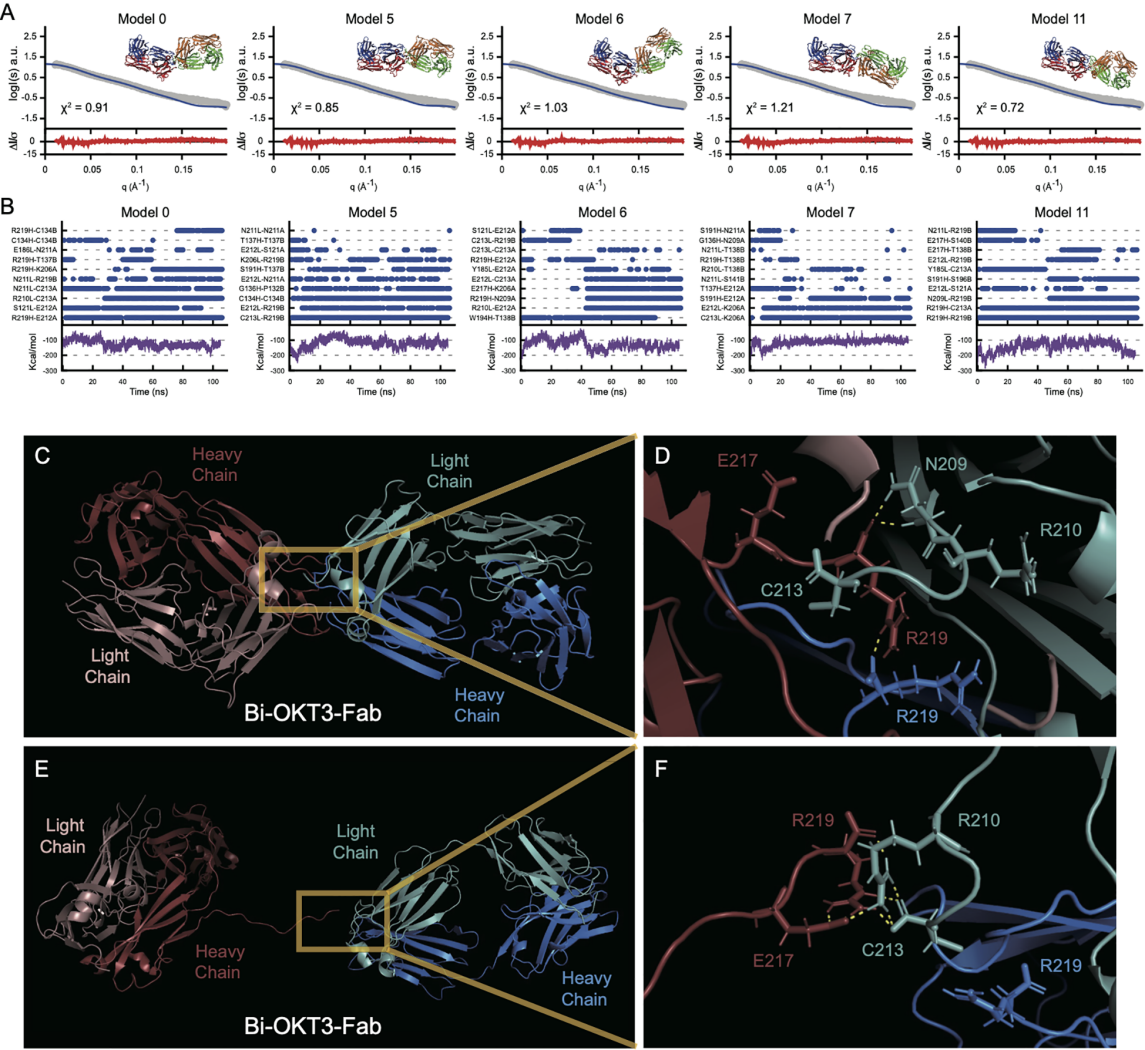
Multiple stably interacting Bi-Fab models fit the experimental SAXS curves. **(A)** the Bi-OKT3-Fab models (model numbers indicated at the top left of each graph) extracted from the 105 ns time point were fitted (blue lines) to the experimental SAXS curve (grey curve) using Foxs fitting program (25). The Bi-OKT3-Fab structures are depicted to the upper right side of each graph, where Fab1 is depicted with red (heavy chain) and blue (light chain) and the Fab2 is depicted with orange (heavy) and light (green). The error weighted residuals are shown at the bottom of each fitting curve in red. **(B)** Multiple interacting amino acid pairs between Fab monomers is required for Bi-Fab stabilization. Individual graphs for Bi-Fab models, identifiers labeled at the top left of each pair of graphs. Upper graphs are a measure of the interaction times of the top eight amino acid pairs and was adapted from Liu et al. (108). Interaction times were determined by breaking the trajectories down into 1 ns intervals and atoms pairs that remained within 2 Å for at least half of the 1 ns interval are given a blue dot at the indicated time intervals. The lower graphs (purple lines) are the linear interaction energies (LIE) between Fab monomers over the entire trajectory and is a sum of the van der Waals and electrostatic interactions using the formula 0.15 * van der Waals + 0.5 * electrostatic interactions. **(C–F)** Example of Bi-OKT3-Fab model 11 interface. **(C)** After 105 ns of MD the tight interface of the right OKT3 (Blue) and left OKT3 (Red). **(D)** Close up of interface showing persistent N209-R219, R219-C213, and R219-R219 interactions. C213 and R219 are the C-terminal residues, and their backbone carboxyl groups participate in contacts. **(E, F)** Example of Bi-Fab model 11 interface after SMD for separation. (E) Retained interaction between Fabs in last frames of the Bi-OKT3-Fab model 11 after 5 ns steered molecular dynamics. **(F)** Close up of interface showing R219-R210, E217-R210, and retained R219 interaction with C213 which greatly hinder Fab separation in Bi-OKT3-Fab model 11.

Next, we used molecular dynamic (MD) simulations to equilibrate these Bi-OKT3-Fab models over a 105 ns trajectory to monitor stability of these non-covalently interacting complexes (75). Electrostatic and van der Waals interaction energy between Fabs was monitored throughout the trajectory and models that dissociated (positive interaction energy) were excluded from further analysis (**Figure 12A**) (76). All eleven Bi-OKT3-Fab models subjected to MD remained associated for the entire simulation (**Figure 12**) proving their stability.

Steered molecular dynamics (SMD) tested potential entanglement in the 11 models being considered by monitoring Fab separation while pulling Bi-Fab constant domains apart. Entanglement results from knots of the protein backbone that prevent separation without covalent bond breakage (77–79) SMD could not separate OKT3 Fabs in Bi-Fab models 1, 2, 3, 9, and 14 revealing their entanglement (**Supplementary Figure S4**). Among these five models, only Bi-OKT3-Fab model 9 could not be separated when the disulfides were broken, showing that four models had disulfide-dependent entangled linked loops (80). Although Bi-OKT3-Fab model 11 did not separate Fabs in SMD (**Figures 12C–F**), it was not entangled. Thus, from the eleven stable models, numbers 0, 5, 6, 7,10, and 11 were not entangled and were studied further.

We next used Bi-OKT3-Fab structures from the MD trajectories to compare to EM and SAXS data. Bi-OKT3-Fab Models 0, 5, 6, 7 and 11 had MD average R_g_ similar to SAXS experimentally determined R_g_ (43.17 +/- 2.33 Å) (**Figures 12**, **10B**). Only Bi-OKT3-Fab model 10 had an MD R_g_ that was far lower and outside the experimental range (**Figures 12**, **10B**). These same five Bi-OKT3-Fab models also fit SAXS intensity curves well (χ^2^< 2.0) (**Figure 12A**) using two different fitting algorithms (**Supplementary Table S5**). Furthermore, these same five models fit the electron density map obtained from OpNS-EM (**Supplementary Table S5**). They all had D_max_ ranging from ~134-147 Å with linear Fab-Fab arrangement with angles ranging from 106-174°. Therefore, Bi-OKT3-Fab models 0, 5, 6, 7, and 11 derived by docking and MD matched experimental EM and SAXS data. From that, these models were considered stably interacting Bi-OKT3-Fab conformers.

### OKT3 Bi-Fabs have less Fab-Fab flexibility compared to F(ab’)2

**Figure 13:**
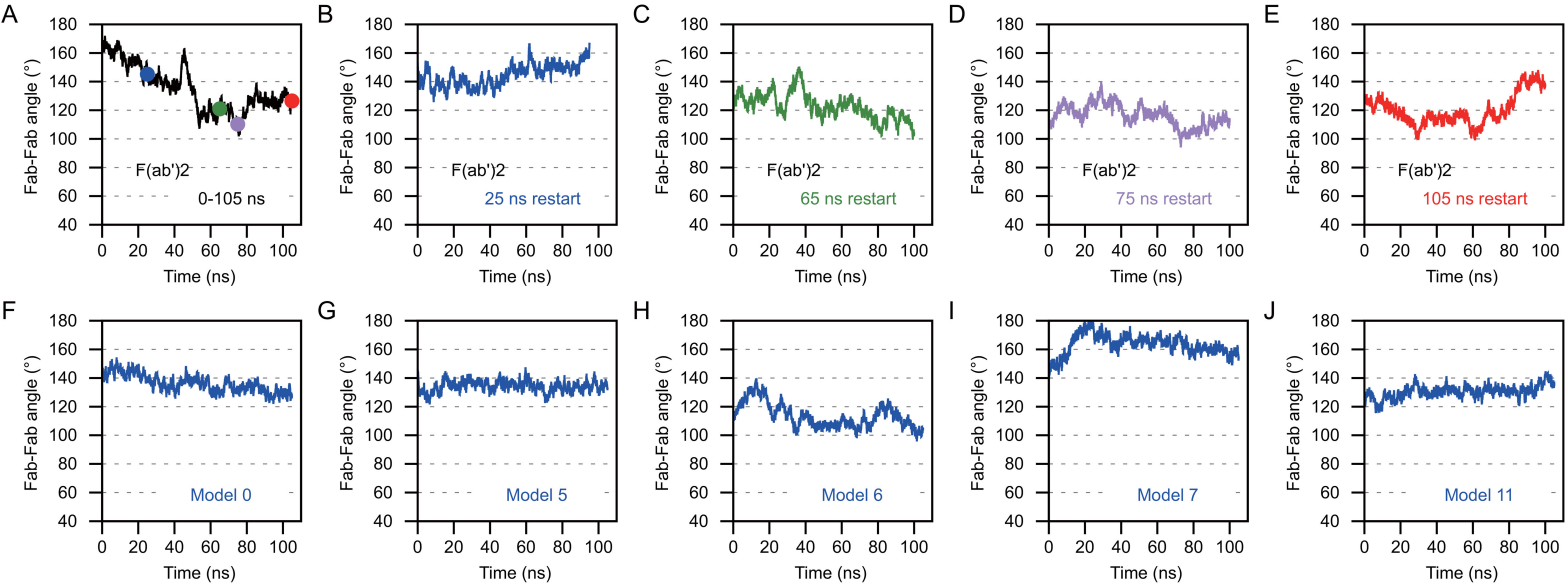
Anti-CD3 Bi-OKT3-Fab display decreased Fab-Fab flexibility than F(ab’)2 Briefly, the Fab-Fab angle was measured by first drawing a vector that extended from the center of mass of the constant domain to the center of mass of the variable domain. This was done for Fab_1_ and Fab_2_ and the angle between the vectors was measured for F(ab’)2 and Bi-Fab. **(A–E)** The Fab-Fab angle measures for OKT3 F(ab’)2 homology model. The F(ab’)2 starting models for B-E were obtained from the indicated time point from the trajectory shown in **(A)** and are color coded according to the time point the starting models originated from. **(F–J)** Fab-Fab angle measurements for the indicated Bi-OKT3-Fab models over a 105 ns trajectory.

Next, we compared the range of Fab-Fab angles that OKT3 Bi-Fabs and F(ab’)2 can sample. To perform this comparison, we equilibrated the OKT3 F(ab’)2 EOM homology model under the same MD simulation conditions as Bi-OKT3-Fab models above. Four frames from the initial 105 ns MD were used to start independent 105 ns MD equilibrations to provide additional samples of F(ab’)2 conformations. The average 147 Å D_max_ of the OKT3 F(ab’)2 model during MD matched that value from SAXS analysis (**Supplementary Table S3**). Additionally, EOM demonstrated that OKT3 F(ab’)2 is very flexible and preferentially adopts Fab-Fab angles of ~110° and ~160°, and this range of Fab-Fab angle sampling can be seen within a 105 ns MD trajectory (**Figures 13A–E**). Under identical conditions, the five Bi-OKT3-Fab models that fit our experimental data indicate that the Fab-Fab angle sampling is more rigid than F(ab’)2 and that they prefer angles of ~130° or between 140° and 160° (**Figures 13F–J**). The standard deviation of Fab-Fab angles for OKT3 F(ab’)2 during 105 ns MD were significantly higher (p=0.026, two-tailed t-test) than that for the Bi-OKT3-Fab angle. This result shows a distinction between stiff linear Bi-Fabs and more flexible F(ab’)2. Fab-Fab angle variation in F(ab’)2 is an expected consequence of flexible linkers that covalently link the two Fabs to the hinge region (**Figure 1**). In contrast, the noncovalent associations of constant domain C-termini in Bi-Fabs thwart such flexibility.

### MD and SMD predict Bi-OKT3-Fab molecules are highly stable and contain a defined interface with residues engaged in Fab-to-Fab stable interactions

Contacts between left and right Fab atoms unveil how Fabs associate in the five best OKT3 Bi-Fab models. We considered atoms to be in non-covalent contact if they are within 2 Å of one another. Plots of inter-Fab contacts during MD revealed several features of Bi-Fab assembly and stability (**Supplementary Figure S5**). First, Fabs interacted via extreme C-terminal residues of heavy and light chains, near residues Arg219 and Cys213, respectively. Second, Fab contacts change from the initial docked model set to a more stable (>40 ns) set during MD. Despite changes in contact set, each Bi-Fab model has a unique set of stable contacts. Third, three or more stable contacts would be sufficient to make stiff Bi-Fabs with small Fab-Fab angle variations and most models had more than this minimum. Fourth, stable, strong contacts used electrostatic interactions.

Bi-Fab model 11 exemplifies stable, strong inter-Fab contacts. SMD, which pulled Fabs apart, separated Fabs in unentangled Bi-Fab models 0, 5, 6, and 7, but failed to separate them in model 11. This model had five stable contacts at the end of 105 ns MD (**Figures 12B–D**; **Supplementary Figure S5**). While Fabs were pulled in Bi-Fab model 11 SMD, some inter-Fab contacts shifted. At the end of model 11 SMD, when other models separated Fabs, electrostatic interactions of left heavy chain Glu217 and Arg219 hung onto right light chain Arg210 and Cys213 (**Figures 12E, F**).

### OKT3 Bi-Fab binds TCR complexes in distinct cells more rigidly compared to F(ab’)2

Next, we made models of Bi-OKT3-Fab bound to two TCR/CD3 complexes. We expected to find models that would enable bridging of T cell to T cells, as requirements for anti-CD3 driven fratricide suggested to happen (**Figures 4**, **5**, **8**). The extracellular and transmembrane domains of the eight subunit TCR/CD3 complex were elucidated by cryo-EM (PDB 6JXR (46)). This complex has two CD3ϵ subunits in CD3ϵδ or CD3ϵγ dimers, which can bind OKT3.The structure of a Fab of OKT3 bound to the CD3ϵγ dimer (PDB 1SY6 (30)) shows how to position OKT3 Fab relative to one of these CD3ϵ subunits. The variable domains of Bi-OKT3-Fab complexes appear capable to bind to two CD3ϵ ectodomains simultaneously, based on the capacity to stimulate human T cells observed for Bi-OKT3-Fab (**Figure 8**). Therefore, we made TCR/CD3-Bi-Fab-TCR/CD3 models by a stepwise procedure using the 6JXR, 1SY6, and our Bi-OKT3-Fab model structures.

**Figure 14:**
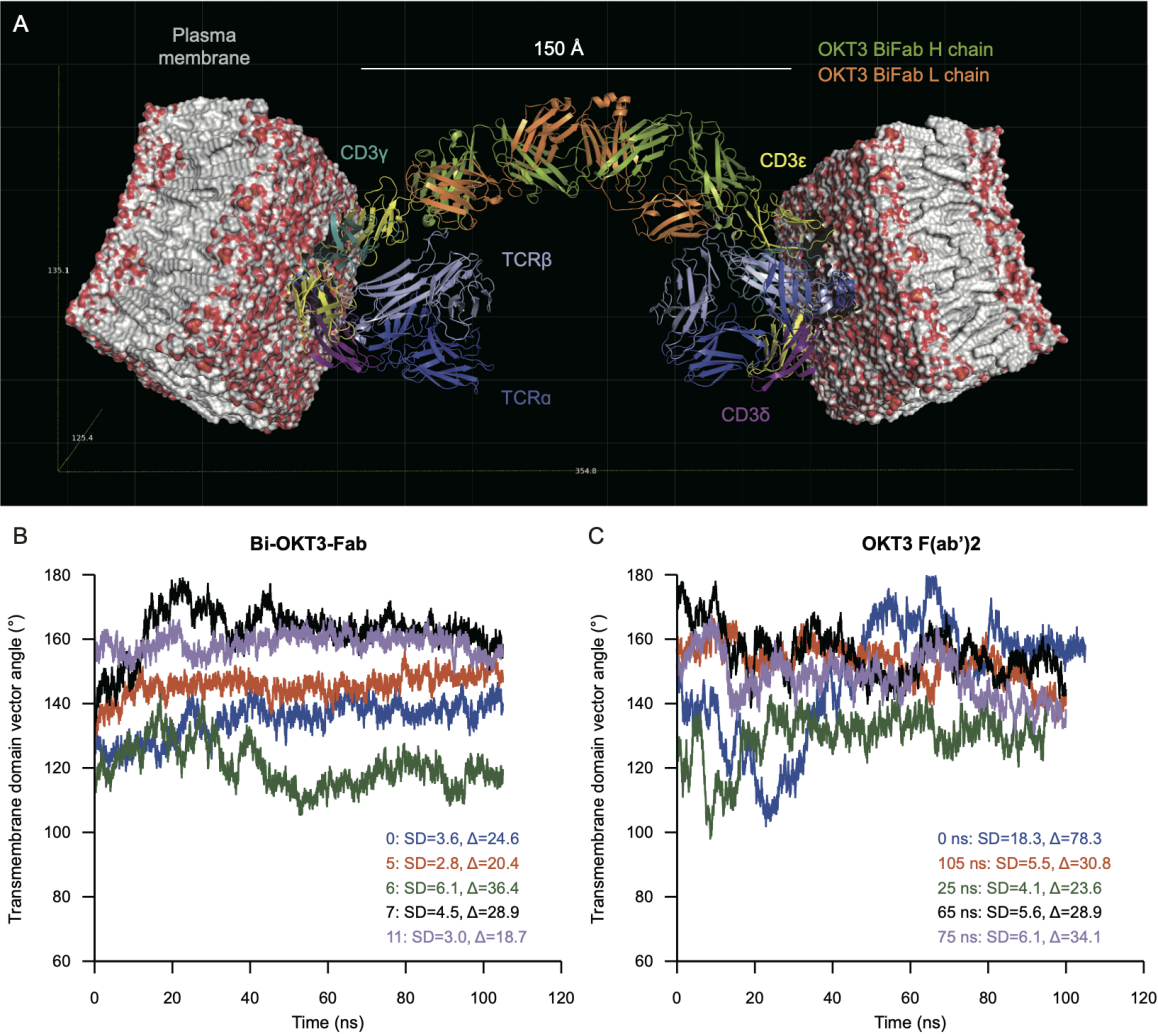
Molecular modeling predicts binding to TCR complexes in distinct cells that is more rigid for Bi-OKT3-Fab than F(ab’)2. **(A)** Docking of Bi-OKT3-Fab model 11 to two human TCR/CD3 complexes (PDB ID: 6XJR) located in two separated T cells, as described in Materials and Methods. Model 11 has the TCR complexes embedded in 100 x 100 Å phosphatidylcholine (POPC) membranes. Bi-OKT3-Fab binds to the CD3εγ subunit in both TCR complexes. Note other binding modes for model 11 to CD3 dimers are compatible, such as Fabs binding to the CD3εδ subunit in both TCR/CD3 complexes or to CD3εγ and CD3εδ subunits in either TCR/CD3 complex. Docking a second Bi-Fab to the available CD3εδ dimer from the T cell membrane on the left side could reach another TCR/CD3 complex located on a different T cell. **(B, C)** Transmembrane vector angles in Bi-OKT3-Fab **(B)** and Bi-OKT3-Fab OKT3 F(ab’)2 **(C)** MD. The standard deviations (SD) and ranges (∆) are from the 20-100 ns intervals of each MD trajectory. The F(ab’)2 trajectories are for the initial (0 ns) or indicated restarts.

Examination of potential TCR/CD3-Bi-OKT3-Fab-TCR/CD3 structures revealed the following. A single Bi-OKT3-Fab complex is incapable of binding to two CD3ϵ chains on the same TCR/CD3 complex. To do so the Bi-OKT3-Fab would need to adopt a U-shaped conformation out of the range of conformers we found for this molecular complex (**Figure 13**). In fact, the way OKT3 Fab binds CD3ϵ, and the almost linear structure of Bi-OKT3-Fab, require the two bound TCR/CD3 complexes to be embedded in membrane regions or domains that are almost parallel to one another. Those TCR/CD3 complexes are most likely in distinct cells slightly over 150 Å apart (**Figure 14A**). In these constructions, the average transmembrane domain vector of the eight TCR/CD3 subunits is perpendicular to the membrane. The angle between two TCR transmembrane domain vectors in TCR/CD3-Bi-OKT3-Fab-TCR/CD3 models was 101° to 155° depending on the Bi-Fab model used. An angle close to 0° would indicate bound TCR/CD3 complexes could be in the same membrane, angles closer to 180° indicates the TCR/CD3-embedded membranes are parallel to one another. Bi-OKT3-Fab model 11, which matched the SAXS data best (**Supplementary Table S5**), had a transmembrane domain vector angle of 155° (**Figure 14B**). Whether Bi-OKT3-Fab bound to CD3ϵ in the CD3ϵδ or CD3ϵγ dimer in the two TCR complexes did not reduce the transmembrane domain vector below 110°for any dimer combination or Bi-OKT3-Fab model. Thus, these TCR/CD3-Bi-OKT3-Fab-TCR/CD3 structures are consistent with the empirical data above describing anti-CD3 Bi-Fabs crosslink distinct T cells to promote their activation and eventual fratricide.

We also observed T cell activation resulting in fratricide by anti-CD3 F(ab’)2 that was less efficient than Bi-Fab. We constructed TCR/CD3-OKT3 F(ab’)2-TCR/CD3 models for comparison seeking a potential molecular basis to the higher stimulatory capacity of Bi-Fabs. These models also showed that OKT3 F(ab’)2 must bind to separate cells to bind to two distinct TCR/CD3 receptors simultaneously. Structures of OKT3 F(ab’)2 from MD frames showed that they produce transmembrane domain vector angles between 100°-180°, a range of 80° (**Figure 14C**). In contrast, the transmembrane domain vector angles for any of the Bi-OKT3-Fab models varied by less than 36.4° (**Figure 14B**). The greater range of OKT3 F(ab’)2 transmembrane domain vector angles compared to Bi-OKT3-Fab suggests that Bi-Fab can hold two T cells in a more rigid configuration. These observations are in line with the comparison of Fab flexibility in ligand-free Bi-Fab and F(ab’)2 shown in **Figure 13**. We speculate this rigidity of the Bi-Fab is responsible for inducing more robust T cell responses when compared to F(ab’)2 and mAb of matching specificity.

## Discussion

Our study strongly suggests potency to stimulate T cells by anti-CD3ϵ mAb, F(ab’)2 and Bi-Fabs, 7D6 and OKT3 depends on the flexibility of the two associated Fabs in each of these molecular complexes. Bi-Fab complexes promoted superior T cell responses compared to F(ab’)2 and mAb (**Figures 2**–**5**). At a structural level, Bi-Fab and F(ab’)2 complexes showed similar linear shapes by EM and SAXS (**Figures 9**–**11**). Molecular docking revealed that Fabs in Bi-Fab complexes associate via their C-termini, with most residues at the Fab interface involved in electrostatic interactions (**Figure 12**). Not only do these linkages promote linear arrangement of CD3ϵ-binding variable regions, but MD shows that the Bi-Fab variable-to-variable linkage is stiffer compared to covalent linkages in the F(ab’)2 complex that include a hinge region. The F(ab’)2 hinge makes a more flexible joint for the two Fabs than the noncovalent interactions in Bi-Fabs. This means that forces imposed at either variable region in Bi-Fab more efficiently transfer displacements to CD3ϵ than F(ab’)2. Physical manipulation can enhance T cell responses from some anti-CD3ϵ antibodies (Kim et al., 2009). Brownian motion that moves Bi-Fab or F(ab’)2 will induce greater CD3ϵ motion from the former because the variable-to-variable linkage is stiffer, making a more efficient lever to move CD3ϵ relative to other TCR subunits. Therefore, greater leverage of CD3ϵ from Bifab induces functional T cell responses such as cell division and cytotoxicity more efficiently than F(ab’)2.

Monovalent engagement of the TCR/CD3 complex with diverse ligands such as soluble peptide/MHC antigens or antibody fragment fails stimulation of T cell function (81, 82). Since anti-CD3 F(ab’)2 and Bi-Fab complexes were competent to induce T cell responses, we expected their variable regions to engage CD3ϵ simultaneously. By using published structures of a human TCR/CD3 complex (46), OKT3 bound to human CD3ϵ (30), and our Bi-Fab structures, we stitched together TCR/CD3-Bi-OKT3-Fab-TCR/CD3 (TCR-Bi-Fab-TCR) assemblies. Constraints at the CD3ϵ to OKT3 junction and between the two Fabs in OKT3 impose two TCR/CD3 complexes bound by Bi-OKT3-Fab to be embedded in adjacent T cell membrane regions, domains, or even separate cells (**Figure 14A**). Therefore, TCR-Bi-Fab-TCR could bring two T cells close together like the immunological synapse of a T cell with an APC. The TCR-Bi-Fab-TCR brings the adjacent membranes to about 180 Å. The immunological synapse brings the T cell and APC membranes within ~150 Å. The T cells attached to the anti-CD3 Bi-Fab increases induction of CD3ϵ forces in both neighboring cells. Activation of two adjacent T cells and potentially greater forces exerted by TCR/CD3 bound cells likely amplifies even small differences in stiffness of F(ab’)2 and Bi-Fab complexes we detected by MD simulations (**Figures 14B, C**).

Our finding that triggering potency of anti-CD3 Fab-based crosslinking reagents may be dictated by the flexibility and angle of Fabs when binding to the TCR/CD3 aligns with a TCR triggering mechanism sensitive to mechanical manipulation. Indeed, one of the current models to explain TCR triggering invokes a pulling force exerted on the receptor (83, 84). In the mechano-sensor model a pull that results in catch-bonds that extend the half-life of TCR/antigen interactions triggers the TCR (85, 86). This force must pull the TCR in a specific direction, making the receptor function as a mechano-sensor (46, 84, 87). When considering this type of trigger, some authors propose that antigen pulling induces conformational changes transmitted from extracellular to cytoplasmic TCR/CD3 domains that eventually enable phosphorylation of ITAMs and subsequent assembly of a CD3 signalosome (88).

Although peptide-MHC ligands presented by APCs are the natural TCR activator, anti-CD3 antibodies also activate T cells and have been used extensively to study TCR/CD3 triggering, signaling, and T cell responses. In fact, first empirical evidence of the TCR functioning as a mechano-sensor was obtained when studying stimulation of T cells with anti-CD3 mAbs binding to different epitopes (84). Productive T cell responses were shown to depend upon where anti-CD3ϵ antibodies bind and the directionality of their subsequent movements (84). These observations were later supported by additional empirical evidence obtained with more sophisticated experimental approaches that use optical tweezers to move anti-CD3ϵ antibodies precisely (87, 89, 90).

Understanding the molecular underpinning of TCR/CD3 triggering by anti-CD3 antibodies is of immediate application for the design of antibody-based therapeutics pursuing activation of immune T cell function by binding to the CD3 complex to activate signal transduction. Anti-CD3 bi-specific antibodies (anti-CD3 BiAbs) are just one example of the multitude of anti-CD3 antibody fragment-based designs that hold promise in the clinic but need improvement to enhance T cell performance (11, 91, 92). The superior stimulatory capacity of the anti-CD3 Bi-Fabs studied here over more flexible, hinge containing variants such as F(ab’)2 and mAb suggest next generations of bi-specific anti-CD3 crosslinkers to include stiffer variable to variable domain linkage connecting T cells with pathological targets as a rational approach to increase the performance of this type of therapeutics. Humanized anti-CD3 Bi-Fab complexes could report clinical benefits over current drugs as therapeutic reagents for depletion of pathological T cell clones.

CD8 T cells display efficient and fast processing of granzymes during their synthesis from the ER towards secretory granules to protect themselves from apoptosis (93). In addition, further resistance to autolysis by perforin/granzyme relays on degranulation pores seemingly functioning unidirectionally towards the target cells (94). However, such mechanisms would not protect from granzymes entering CD8 T cells targeted by CTLs, as observed previously in different experimental models (94), and in this study when crosslinking T cells via anti-CD3 IgGs. Interestingly, a very recent publication describes cell softness found in CD8 T cells as unsupportive of mechanical forces required for efficient degranulation onto targets. Authors invoke cell softness regulated by cytoskeleton protein filamin A to explain CTLs avoiding perforin-mediated autolysis, and to explain CD8s as inefficient targets of CTLs (95). Indeed, since Bi-Fabs appear as the most rigid CD3 crosslinker of the anti-CD3 IgG molecules we have compared, they could rigidify the plasma membrane of crosslinked T cells further for superior perforin/granzyme lysis when CTLs are involved in T cell fratricide.

Presently, the use of Fc-containing antibody therapeutics that target human T cells for their neutralization (by steric hindrance over TCR/antigen recognition) and depletion (by Antibody Dependent Cell-mediated Cytotoxicity (ADCC) and/or Complement Dependent Cytotoxicity (CDC)), such as muromonab (Ms IgG OKT3, FDA approved to treat acute graft rejection) (96) or teplizumab (humanized OKT3 lacking FcR binding capacity), FDA approved to delay onset of Type I Diabetes (T1D) (97, 98) also cause undesired side effects due to remaining capacity of the Fc portion of these antibodies to support crosslinking of the CD3 complex and activation of T cells (66, 99). The potential occurrence of these side effects, although minimized in the case of teplizumab by reducing TCR crosslinking capacity when impairing Fc binding to Fc receptors, requires strict patient monitoring, and in some cases calls for treatment discontinuation. Thus, there is a need in the field for novel strategies to prevent these side effects, also observed in the case of bi-specific T cell engaging antibodies and CAR T cells (100, 101). Since Bi-7D6-Fab surpassed 7D6 F(ab’)2 and mAb capacity to drive T cells into fratricide while overriding cell proliferation *in vitro* (**Figures 2**, **3**, **4**), as well as *in vivo* in the absence of cytokine release syndrome (**Supplementary Table S2**), developing recombinant anti-TCR and/or anti-CD3 Bi-Fabs functionally bivalent might deliver a more efficient and safer strategy to remove pathogenic T cells when aiming to prevent progression of autoimmune diseases such as T1D, or graft rejection upon organ transplantation, than current anti-CD3 Fc-containing Igs. Additional research, using pertinent pre-clinical animal models, will be required to compare side by side these strategies for their efficacy to deplete T cells and their toxicity.

We observed formation of Bi-Fabs in papain digestions of IgG mAbs from multiple species (Mouse IgG2a, kappa: 7D6, OKT3; Armenian Hamster IgG: 2C11, H57 (16). H146 (unpublished); Rat IgG2b, kappa:17A2 (16). These observations suggest Ig domains could have been under selective pressure to conserve sequences that permit Fab dimerization. Some bacteria evade antibodies by digesting them into Fabs, F(ab’)2 and/or variable domains (102–106). During an infection by bacteria with such evasion mechanisms, dimerization of released Fabs into Bi-Fabs could reestablish more efficient epitope neutralization. In fact, residues in the constant domain of the mouse IgG heavy chain with high interaction frequencies in the Fab/Fab interface of Bi-OKT3-Fab according to our MS models (**Figure 12B**) are conserved between mouse, human and rat IgG heavy chains subtypes (**Supplementary Figure S6**). Though we have only observed *in vitro* Bi-Fab formation when digesting IgGs from different species that carry kappa light chains, the lambda chains from different species present conserved residues with the kappa isoforms at positions identified by molecular dynamic simulation to interact at the interface between the two Fabs (**Supplementary Figure S6**). Thus, we expect Bi-Fab formation from monoclonal IgG and A isotypes from the species aligned. Further studies to determine whether Bi-Fab formation may happen *in vivo*, in the context of infection with bacteria digesting antibodies into Fabs, and the potential effects of such Bi-Fabs to control the pathogen are needed to determine a potential physiological function of Bi-Fabs.

In summary, by comparing stimulatory capacity of different bivalent anti-CD3 crosslinking molecules (mAb, F(ab’)2, Bi-Fab) we observed more robust T cell responses when crosslinking the TCR/CD3 complex with bivalent Bi-Fabs that bind CD3 with less flexibility within the Fabs than in F(ab’)2 and mAb molecules. These findings contribute to better understanding of the requirements for productive TCR/CD3 triggering when targeting the CD3 complex with Fab-containing reagents. Also, they suggest potential strategies to increase potency of current bi-specific anti-CD3 therapeutics by introducing rigidity in their molecular design, as well as ways to decrease toxicity of mono-specific anti-CD3 therapeutics such as teplizumab by removing Fc. Finally, our observations pose the intriguing possibility that Fab dimerization by a non-covalent interface revealed upon hinge and Fc removal is a conserved feature across Igs that could serve to re-establish Fab bivalence upon degradation by pathogens.

## Data Availability

The original contributions presented in the study are included in the article/supplementary materials. Further inquiries can be directed to the corresponding authors.
