# Supplementary material for: Rigid crosslinking of the CD3 complex leads to superior T cell stimulation
Source: Front Immunol. 2024 Aug 30;15:1434463. doi: 10.3389/fimmu.2024.1434463 (PMC11392757; doi:10.3389/fimmu.2024.1434463)
Supplement: Supplementary file 1 [file DataSheet1.pdf]

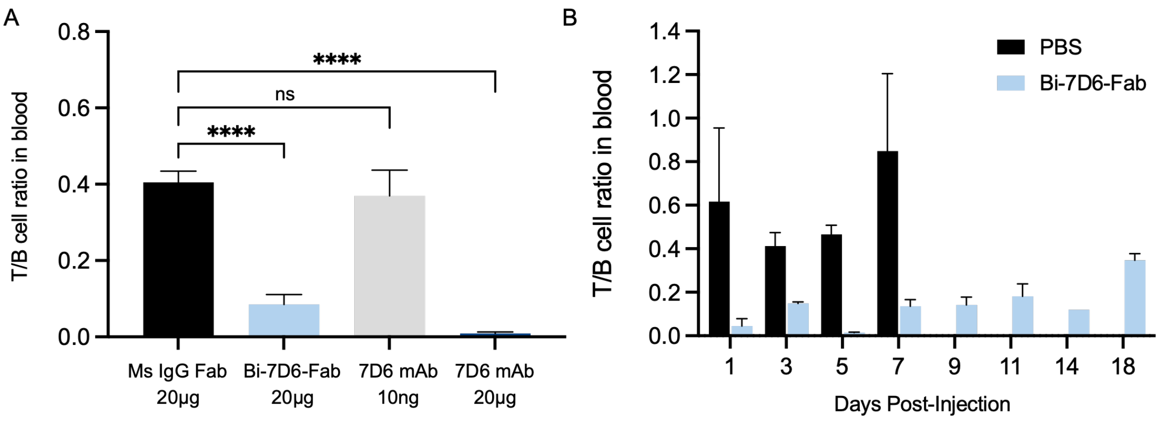

Figure S1. Intravenous administration of Bi-7D6-Fab reduces the frequency of T cells in peripheral blood.

(A) B6 mice reported in Supplemental Table 2 were i.v. injected in groups of three with either 20 µg of Ms IgG Fab, Bi-7D6-Fab or 7D6 mAb, or with 10 ng of 7D6 mAb, to be bled 24 h later for flow cytometry analysis of the ratio of live T to B cells found in PBMC samples. **(B)** Groups of three B6 mice were i.v. injected three consecutive times every 48 h with either PBS or 20 µg of Bi-7D6-Fab to be bled every other day in between injections and up to day 18 for flow cytometry analysis of the ratio of live T to B cells found in PBMC samples. T cells were gated as PI- Thy1.2+ B220-. B cells were gated as PI- Thy1.2- B220+. Error bars represent +/- SE from replica mice. One-way ANOVA test (ns $p > 0.05$, * $p \leq 0.05$, ** $p \leq 0.01$, *** $p \leq 0.001$, **** $p \leq 0.0001$)

A Constant Light Chain Domain

```
7D6       SSEQLTSGGASVVCFLNNFYPKDINVKWKIDGSERQNGVLNSWTDQDSKDSTYSMSSTLT  -180
OKT3      SSEQLTSGGASVVCFLNNFYPKDINVKWKIDGSERQNGVLNSWTDQDSKDSTYSMSSTLT  -179
          ************************************************************

7D6       LTKDEYERHNSYTCEATHKTSTSPIVKSFNRNEC   181-214
OKT3      LTKDEYERHNSYTCEATHKTSTSPIVKSFNRNEC   180-213
          **********************************
```

B Constant Heavy Chain Domain

```
7D6       KTTAPSVYPLAPVCGDTTGSSVTLGCLVKGYFPEPVTLTWNSGSLSSGVHTFPAVLQSDL 180
OKT3      KTTAPSVYPLAPVCGGTTGSSVTLGCLVKGYFPEPVTLTWNSGSLSSGVHTFPAVLQSDL 180
          ***************.********************************************

7D6       YTLSSSVTVTSSTWPSQSITCNVAHPASSTKVDKKIEPRG 220
OKT3      YTLSSSVTVTSSTWPSQSITCNVAHPASSTKVDKKIEPR- 219
          ***************************************
```

Figure S2. Alignment of constant domains of heavy and light chains of 7D6 and OKT3 IgGs

OKT3 (PDB ID: 1SY6) and 7D6 constant domain (Uniprot Accession: P01865) sequence alignments for the **(A)** light chain and **(B)** heavy chain. Sequence alignment shows nearly identical constant domains.

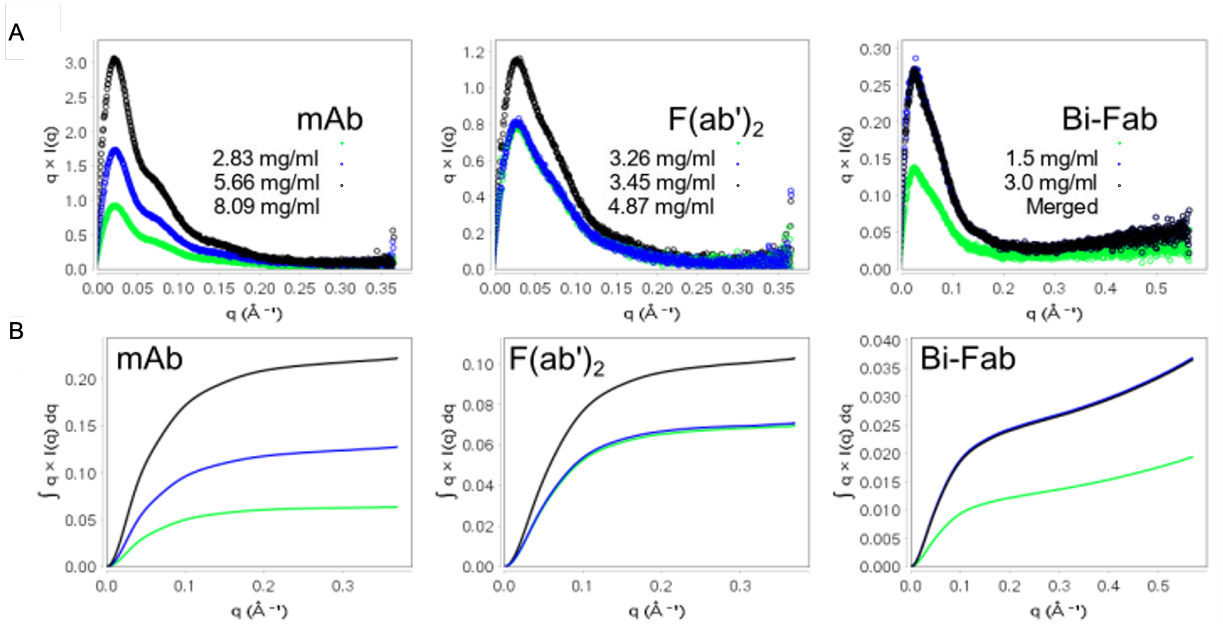

Figure S3. SAXS buffer subtraction
(A) Product of q*I(q) vs q for the mAb (left), F(ab')2 (center), and Bi-Fab (right) under different concentrations. **(B)** Numerical integration of data shown in panel A vs q. The plateaus for mAb and F(ab')2 indicate good buffer subtraction. However, Bi-OKT3-Fab failed to plateau, which compromised using EOM analysis for that antibody.

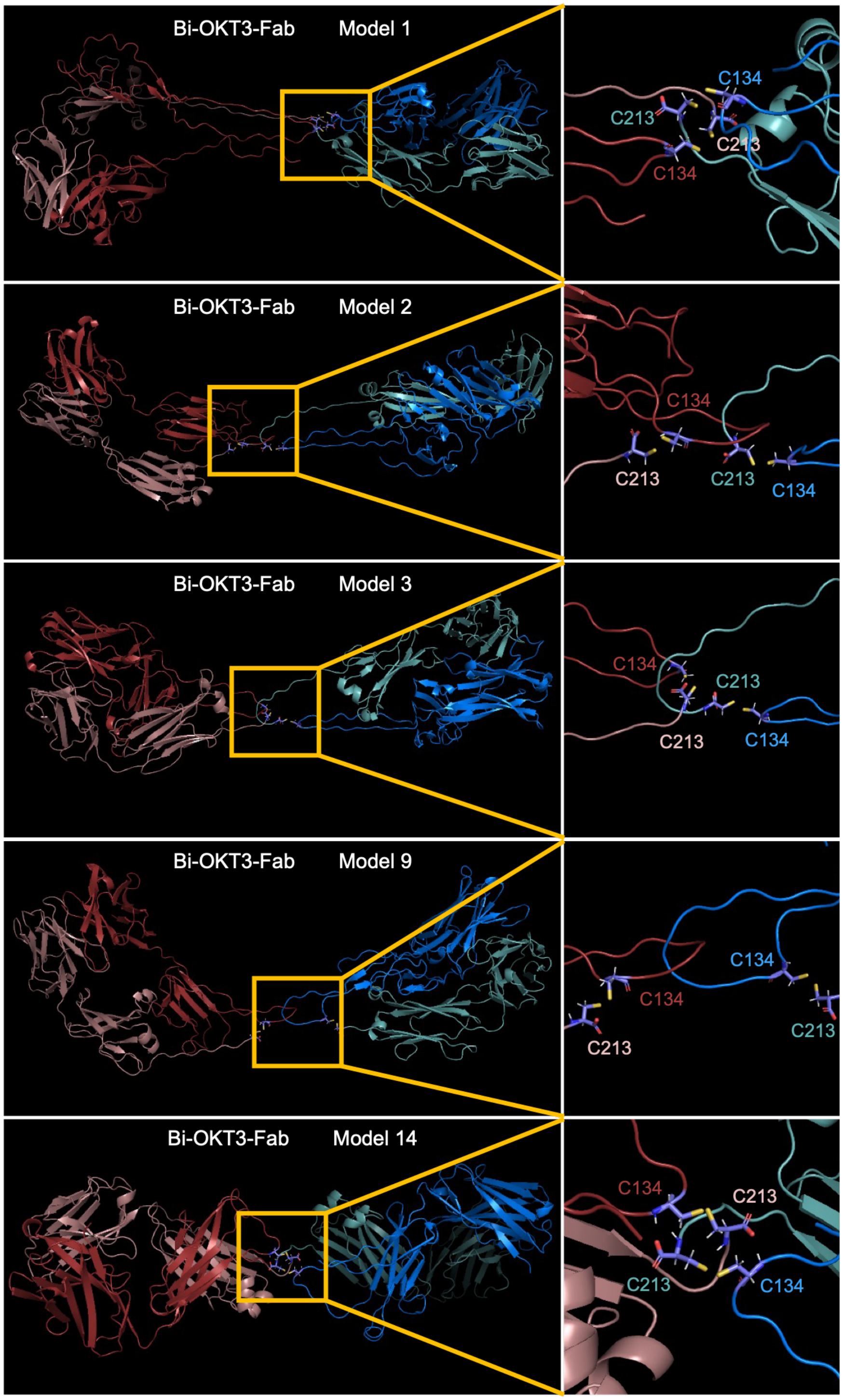

Figure S4. Entangled Bi-Fab models after constant domains were pulled 50 Å apart by SMD.
Models are color coded for Left OKT3 heavy chain (red), left OKT3 light chain (pink), right OKT3 heavy chain (blue), right OKT3 light chain (cyan). Cysteine residues that form entanglement loops are labeled in the enlarged right panels with the same color code.

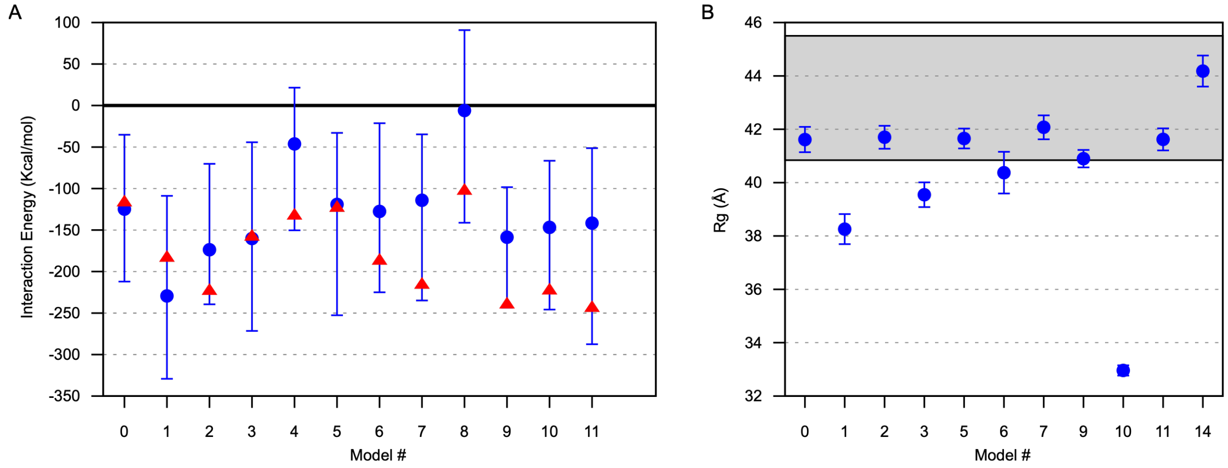

Figure S5. Identification of stably interacting models for Bi-OKT3-Fab.
(A) Fab-Fab interaction energies for Bi-OKT3-Fab models. Shown are the minimum (upper horizontal bar), maximum (lower horizontal bar), average (blue dot), and the starting (red triangle) Fab-Fab interaction energies. **(B)** Average radius of gyration (Rg) for stably interacting Bi-OKT3-Fab models through constant-constant domains for over a 105 ns trajectory. Shaded area indicates the Rg range obtained from the SAXS data.

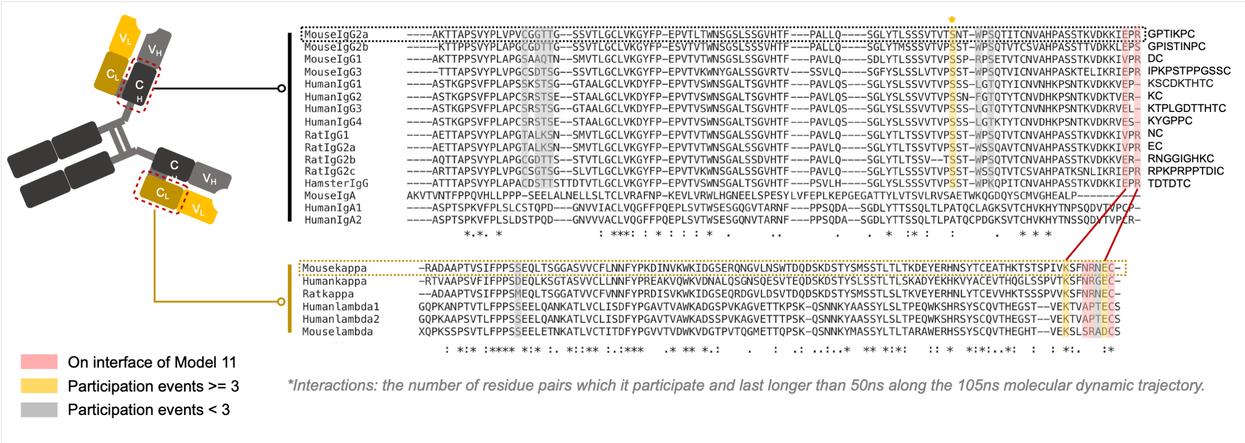

Figure S6. Constant Fab sequence alignment of various isotype of antibodies between rodents and human

Constant Fab sequence of both heavy and light chains are aligned. Sequence of Mouse IgG2a kappa (as the antibody subtype for OKT3 and 7D6) is listed at the top of each alignment. Sequence alignments are performed with all known IgG subtypes of human and common rodents. The known IgA sequences of mouse and human are listed to compare the conservation between isotypes. Sequences are the downstream of Fab C-termini are listed separately end up with the first N-termini cysteine of the respective hinge region, to indicate the range of potential papain digestion sites. According to the result of molecular dynamic simulation, potentially crucial residues involved in Fab-Fab interaction are highlighted with different colors based on the location and number of interactions*. Residue pairs involved in potential electrostatic interaction between heavy and light chains (Glu^{H}-Lys^{L}, Arg^{H}-Glu^{L}) are indicated as red link. Ser191 in heavy chain which contributes to multiple hydrophilic interaction in MD models are indicated with star.

	OKT3 mAb	OKT3 F(ab')2	OKT3 Bi-Fab
Number of residues	1316	888	864
MW based on a.a. seq, Da	146,300	96,550	94.340
Loading concentration, mg/ml	2.83, 5.66, 8.09	3.26, 3.46, 4.87	0.75, 1.5
SAXS data collection	SEC-SAXS*	SEC-SAXS*	HT**
Injection volume, ml	60	60	30
Flow rate, ml min^{-1}	0.5	0.5	N/A
Temperature, K	298	298	283
SEC column	Shodex KW803	Shodex KW803	N/A
Beamline	ALS SIBYLS 12.3.1	ALS SIBYLS 12.3.1	ALS SIBYLS 12.3.1
Beam energy, keV	12	12	11
Sample-detector distance, m	1.5	1.5	1.5
Exposure time, s	3	3	0.3
Images	600	600	32
q range, ($Å^{-1}$)	0.007-0.369	0.007-0.369	0.01-0.3

* SEC-SAXS: Size exclusion SAXS
** HT: high throughput SAXS

Table S1: SAXS collection parameters.

Cytokine Release Syndrome Symptom	Observations
Piloerection	Absent in all treatments
Diarrhea	Absent in all treatments
Hypoglycemia	Absent in all treatments
TNF-α	n.d. in blood serum samples from all treatments
IL-1β	n. d. in blood serum samples from all treatments

Table S2. Intravenous administration of Bi-7D6-Fab does not cause CRS symptoms.
B6 mice were injected in groups of three with either 20 µg of Ms IgG Fab, Bi-7D6-Fab or 7D6 mAb, or with 10 ng of 7D6 mAb. Mice were monitored at time 0, 3, 5 and 24 h post-injection for piloerection and diarrhea. Blood samples were collected at those time points to measure glycemia and detect cytokine secretion (0, 3h, 5h, 24h).

Sample	Conc. (mg ml^{-1})	I(0)^G (S.D.)	Rg^G (Å^{-1}) (S.D.)	I(0)^P (S.D.)	Rg^P (Å^{-1}) (S.D.)	D_{max} (Å)	Vp^P (Å^3)	MW (kDa) (rel to MW_{seq})
OKT3 mAb	2.83	59.62 (0.45)	50.37 (5.04)	59.67 (0.30)	50.76 (0.23)	163	250,907	161.5 (1.10)
	5.66	110.78 (0.51)	48.62 (2.58)	112.50 (0.26)	49.93 (0.09)	153	245,625	174.1 (1.19)
	8.09	203.32 (0.78)	50.19 (4.20)	202.2 (0.36)	50.18 (0.90)	153	246,737	172.5 (1.18)
OKT3 F(ab')2	3.26	43.89 (0.30)	41.18 (4.12)	45.10 (0.21)	43.81 (0.22)	150	118,525	96.0 (0.99)
	3.45	43.20 (0.37)	41.61 (6.04)	44.22 (0.24)	43.92 (0.28)	147	118,277	94.5 (0.98)
	4.87	62.89 (0.23)	41.22 (4.65)	64.37 (0.20)	43.50 (0.15)	145	116,978	94.1 (0.97)
Bi-OKT3-Fab	0.75	7.58 (0.08)	43.17 (2.33)	7.63 (0.08)	44.84 (0.50)	146	103,000	78.9 (0.84)
	1.5	15.73 (0.22)	44.91 (3.44)	15.45 (0.18)	45.12 (0.51)	149	106,000	80.3 (0.85)
	merge	15.05 (0.16)	43.17 (2.33)	15.16 (0.16)	44.92 (0.51)	147	104,404	79.7 (0.84)

Table S3: SAXS derived biophysical parameters for OKT3 mAb, F(ab')2 and Bi-Fab. I(0)^G (S.D.) and Rg^G (S.D.) were obtained from Guinier plot: ln[I(q)] vs q^2 (*66-68*). I(0)^P (S.D.), Rg^P (S.D.) and Vp^P were obtained from Porod plot: (ln[I(q)] vs ln(q)) (*69*). MW was evaluated by SAXS-MOW2 using q max limit of 8/Rg (109). MW evaluation based on aminoacid sequence was obtained using ExPASy (110).

Cluster	Cluster size	Overall Occurrence (%)	Rg (S.D), Å	Dmax (S.D.), (Å)	Fab-Fab angle (°)
OKT3 mAb					
14	17	14.0	52.2 (0.0)	155.42 (0.0)	135
16	22	18.2	54.4 (0.0)	163.44 (0.0)	134
2	1	0.8	54.4	166.54	132
18	26	21.5	50.3 (0.0)	157.81 (0.0)	131
7	5	4.1	50.2 (0.0)	150.5 (0.0)	128
9	2	1.7	48.1 (0.0)	149.57 (0.0)	124
4	2	1.7	51.3 (0.0)	147.47 (0.0)	121
10	4	3.3	51.0 (0.0)	155.59 (0.0)	119
13	3	2.5	50.3 (0.0)	154.58 (0.0)	117
3	1	0.8	49.9	149.8	115
8	1	0.8	53.9	163.83	114
11	2	1.7	54.0 (0.0)	167.81 (0.0)	106
5	4	3.3	48.2 (0.0)	162.49 (0.0)	95
17	17	14.0	50.5 (0.0)	161.68 (0.0)	83
6	1	0.8	50.9	156.15	71
1	1	0.8	51.0	156.87	70
15	9	7.4	50.6 (0.0)	157.5 (0.0)	70
12	3	2.5	53.3 (0.0)	168.29 (0.0)	41
Total	121				
OKT3 F(ab')2					
16	13	10.0	51.6 (1.0)	161.9 (2.2)	161
13	17	13.1	51.2 (0.9)	161.2 (2.3)	159
7	5	3.8	52.7 (0.4)	163.6 (0.9)	157
10	8	6.2	43.5 (0.8)	138.2 (2.1)	137
19	9	6.9	40.9 (1.0)	131.2 (4.3)	137
5	13	10.0	44.4 (0.0)	139.4 (0.0)	110
17	19	14.6	45.0 (0.5)	140.4 (1.1)	108
14	2	1.5	39.7 (0.0)	128.4 (0.0)	101
11	5	3.8	44.3 (0.7)	138.6 (1.9)	100
1	1	0.8	43.2	139.5	98
6	2	1.5	43.4 (0.0)	135.7 (0.0)	97
8	5	3.8	43.5 (0.7)	136.8 (2.8)	79
3	1	0.8	43.0	135.1	75
2	5	3.8	37.5 (4.7)	115.1 (4.7)	73
18	2	1.5	40.3 (2.3)	127.5 (7.1)	69
12	6	4.6	38.7 (1.3)	116.6 (4.4)	62
9	3	2.3	37.8 (1.3)	117.1 (4.8)	49
4	4	3.1	37.4 (0.3)	111.0 (0.3)	39
15	10	7.7	37.7 (0.9)	114.5 (2.0)	31
Total	130				

Table S4: Shape clustering of the structures from optimized ensembles using DAMCLUST

Model	EM density map correlation	Volume ($Å^3$)	D_{max} (Å)	R_g (Å) (S.D.)	Fab-Fab angle, ° (S.D.)	$\chi2^{Cry}$	$\chi2^{Fo}$
0	0.9035	163,500	136	41.61 (0.47)	136 (6)	1.93	0.91
5	0.9147	165,100	141	41.65 (0.37)	134 (4)	1.61	0.85
6	0.8983	163,800	139	40.37 (0.78)	113 (9)	1.89	1.03
7	0.8671	159,700	144	42.07 (0.45)	163 (7)	2.27	1.21
10	0.8057	152,000	109	32.96 (0.19)	66 (6)	9.57	7.28
11	0.8718	163,900	142	41.62 (0.41)	130 (4)	1.26	0.72

Table S5: Biophysical parameters of Bi-OKT3-Fab models.
In EM density map correlation, simulated maps of Bi-OKT3-Fab models (105 ns) obtained from MD simulations at a resolution of 20Å were fitted into OpNS-EM density map using Chimera. Volume and D_{max} are values obtained from the 105 ns Bi-OKT3-Fab models using Crysol. Rg (S.D.) and Fab-Fab angle (S.D.) indicates average and standard deviations correspondingly obtained from 0-105ns MD trajectory of Bi-OKT3-Fab models. $\chi2^{Cry}$ and $\chi2^{Fo}$ are SAXS fitting using Crysol or FoXS (*25*) respectively.

Model	Fab-Fab angle, ° (S.D.)
0-105 ns	135 (16)
25 ns restart	120 (11)
65 ns restart	144 (8)
75 ns restart	123 (9)
105 ns restart	116 (8)

Table S6: Biophysical parameters of OKT3 F(ab')2 models.
Fab-Fab angle (S.D.) indicates average and standard deviations correspondingly obtained from 0-105ns MD trajectory of OKT3 F(ab')2 models.

Supplementary References:

109. V. Piiadov, E. Ares de Araújo, M. Oliveira Neto, A. F. Craievich, I. Polikarpov, SAXSMoW 2.0: Online calculator of the molecular weight of proteins in dilute solution from experimental SAXS data measured on a relative scale. *Protein Science* **28**, 454–463 (2019).
110. E. Gasteiger, ExPASy: the proteomics server for in-depth protein knowledge and analysis. *Nucleic Acids Res* **31**, 3784–3788 (2003).
